# Not as Ubiquitous as We Thought: Taxonomic Crypsis, Hidden Diversity and Cryptic Speciation in the Cosmopolitan Fungus *Thelonectria discophora* (Nectriaceae, Hypocreales, Ascomycota)

**DOI:** 10.1371/journal.pone.0076737

**Published:** 2013-10-18

**Authors:** Catalina Salgado-Salazar, Amy Y. Rossman, Priscila Chaverri

**Affiliations:** 1 Department of Plant Science and Landscape Architecture, University of Maryland, College Park, Maryland, United States of America; 2 Systematic Mycology and Microbiology Laboratory, Agriculture Research Service, United States Department of Agriculture, Beltsville, Maryland, United States of America; University of Sydney, Australia

## Abstract

The distribution of microbial species, including fungi, has long been considered cosmopolitan. Recently, this perception has been challenged by molecular studies in historical biogeography, phylogeny and population genetics. Here we explore this issue using the fungal morphological species *Thelonectria discophora,* one of the most common species of fungi in the family Nectriaceae, encountered in almost all geographic regions and considered as a cosmopolitan taxon. In order to determine if *T. discophora* is a single cosmopolitan species or an assemblage of sibling species, we conducted various phylogenetic analyses, including standard gene concatenation, Bayesian concordance methods, and coalescent-based species tree reconstruction on isolates collected from a wide geographic range. Results show that diversity among isolates referred as *T. discophora* is greatly underestimated and that it represents a species complex. Within this complex, sixteen distinct highly supported lineages were recovered, each of which has a restricted geographic distribution and ecology. The taxonomic status of isolates regarded as *T. discophora* is reconsidered, and the assumed cosmopolitan distribution of this species is rejected. We discuss how assumptions about geographically widespread species have implications regarding their taxonomy, true diversity, biological diversity conservation, and ecological functions.

## Introduction

The high plasticity of morphological characters in fungi led early taxonomists to group or “lump” similar-looking species into one individual species. Accordingly, as this species was found in a wide geographic range, it was then labeled as being cosmopolitan *i.e.,* having a worldwide distribution. This resulted in long-held assumptions about long-distance dispersal capabilities in fungi [1]–[2], a trend that was also assumed for other microorganisms such as protozoa, protophytes, and other small organisms (*i.e.,* those less than 1 mm in length such as nematodes, rotifers and marine invertebrates) [3]–[7]. Assumptions about long-distance dispersal, or the well-known Bass-Becking hypothesis [6]–[9], implied that propagules could be easily carried by wind or water so they can be distributed everywhere. However these mechanisms of long-distance dispersal, as well as patterns of geographical distribution and factors driving species diversity, are less known for microorganisms when compared with those of macroorganisms, and have resulted in a drastic underestimation of the global species richness of microorganisms and fungi [10]–[11]. Cosmopolitanism also implies that the number of species has remained stable through time and is relatively small with virtually nonexistent extinction episodes [7], [12]. These are phenomena commonly seen at the family and genus levels; however, at the species level, many cases were cosmopolitanism has been assumed have been disproven by molecular data [5]. While some empirical studies have supported the Bass-Becking hypothesis in some species of microorganisms [6], [12]–[15], there are many examples, not only for the majority of fungi [2], [16]–[21], but also for other microbial eukaryotes, marine and fresh water invertebrates, and even for some protists [22]–[27], where this hypothesis has been disproven. Since the rise of molecular phylogenetics over two decades ago, the view that microorganisms have large-scale spatial distributional ranges has changed, mostly due to the use of molecular markers and phylogenetic analyses, which have increased the level of resolution in systematics studies [2]. Studies have found that there is a distinctive correlation between geographical distance and similarity in community composition (distance-decay relationship) observed in local to global-scale analyses [9].

**Figure 1:**
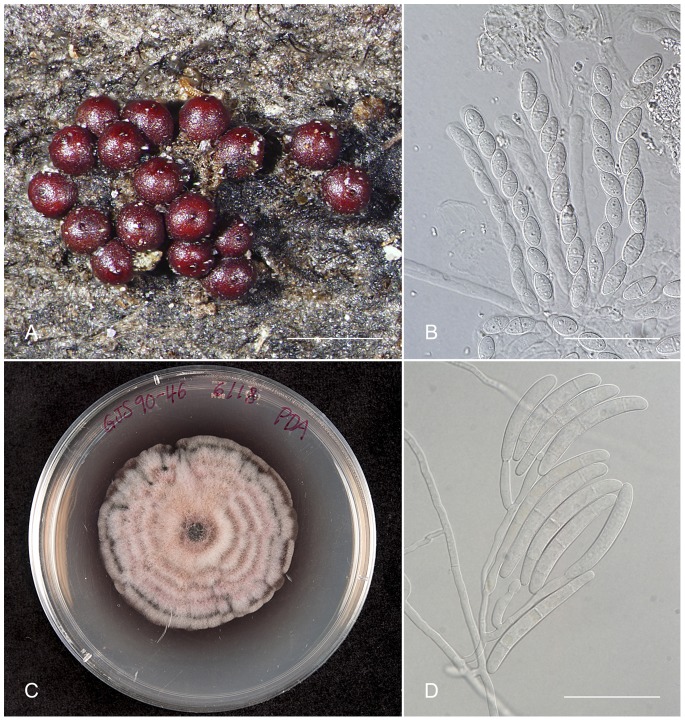
Typical morphology of *T. discophora*-like species. A. Perithecia (sexual fruiting bodies). B. Asci and ascospores (sac and sexual spores). C. Aspect of colony on PDA (potato-dextrose agar). E. Conidiophores and conidia (asexual structures and spores). Bars A = 500 µm, B = 50 µm, D = 50 µm.

In the present study, we explore various issues concerning cosmopolitanism and conservative application of species names and the consequences these bring to taxonomy, true diversity estimates, biological species conservation efforts and ecological functions. We focused our study on *Thelonectria discophora* (Mont.) P. Chaverri & C. Salgado (2011), one of the most representative and common species of the fungal family Nectriaceae (Hypocreales, Ascomycota) and the type species of the genus *Thelonectria*. *Thelonectria discophora* is a saprobe on newly dead, organic plant material where it is among the first colonizers [28]–[31]. It is common in disturbed areas with recently fallen or cut plant material and is rarely found in old growth forests [32], thus serving as an indicator of forest disturbance. This species has been reported to have a cosmopolitan distribution, being encountered on every continent, excluding Antarctica and the Arctic regions [28]–[31]. It is found in a diverse set of habitats on plant substrata such as on the bark of twigs and branches or trunks of recently dead or dying trees, with little morphological variability (Figure 1) [28]–[30], [33]–[34]. This species has also been reported as the causal agent of a distinctive basal canker of cultivated *Rubus idaeus* and *R. fruticosus*. However, it has been regarded as a secondary or weak pathogen since disease outbreaks have been mostly correlated with stressed plants following wind damage or waterlogging [28], [35].

To determine if *T. discophora* is truly cosmopolitan or a complex of geographically and ecologically restricted species, we conducted a series of multi-locus phylogenetic and genetic divergence analyses. We studied representatives of *T. discophora*-like fungi, including taxa considered synonyms of *T. discophora,* collected from a wide geographic distribution. Data were subjected to Bayesian Inference and Maximum Likelihood analyses using 6-gene nuclear DNA sequence data sets. We compared the results of the phylogenetic analyses using standard gene concatenation with those of Bayesian Concordance analyses [36] and Bayesian Inference of Species Trees [37]. Combining these methods allows us to assess whether the genetic divergence among isolates in *T. discophora* is sufficient to consider them as separate units, and to obtain the concordance phylogeny and species tree that best describes the diversity of putative species in this complex.

## Materials and Methods

### Fungal Isolates

A total of 66 isolates from different localities and hosts with morphology corresponding to *T. discophora*, including some with names that have been considered synonyms, were included in this study (Table S1, S2). Five isolates representing *T. lucida* (Höhn.) P. Chaverri & Salgado (2011) were used as outgroup in the phylogenetic analyses. Specimens and cultures were obtained from CABI Bioscience (IMI); Centraalbureau voor Schimmelcultures (CBS); Japanese Ministry of Agriculture, Fisheries and Food Collection (MAFF); New York Botanical Garden (NY); and U.S. National Fungus Collection (G.J.S, A.R).

### Ethics Statement

No permits were required for the described study, which complied with all relevant regulations. This study did not involved endangered or protected species.

### Polymerase Chain Reaction, Sequencing, Alignment and Data Compatibility

DNA extraction and PCR protocols were carried out as described by Chaverri *et al.* [34]. Six nuclear loci were sequenced for this study: partial large nuclear ribosomal subunit (LSU, *ca*. 900 bp), complete internal transcribed spacers 1 and 2 (ITS, including 5.8S of the nuclear ribosomal DNA, *ca*. 600 bp), partial β-tubulin (*tub*, *ca*. 500 bp), α-actin (*act*, *ca*. 600 bp), RNA polymerase II subunit 1 (*rpb1*, *ca*. 700 bp), and translation elongation factor 1α (*tef1*, *ca*. 700 bp) (Table S3). These nuclear loci are commonly used for phylogenetic studies of fungi in the order Hypocreales proving useful for species level studies [34], [38]. Clean PCR products were sequenced in both directions at the University of Maryland DNA Sequencing Facility (Center for Agricultural Biotechnology, University of Maryland, College Park, Maryland, U.S.A.). Sequences were assembled and edited using the program Sequencher 4.9 (Gene Codes, Madison, Wisconsin, U.S.A.).

Alignments were performed using MAFFT version 6 (http://mafft.cbrc.jp/alignment/server/) using the e-ins-i strategy [39] or PRANK [40] implemented by The GUIDANCE Server (http://guidance.tau.ac.il/index.html) [41], using default settings. The PRANK algorithm was used especially for multiple sequence alignments of nuclear loci with rapidly evolving regions and high incidence of insertions and deletions, such as *tef1*, *tub*, *rpb1* and ITS. This method is used to treat insertions correctly and avoid over-estimation of the number of deletion events in the alignments [40]. The program Concaterpillar v. 1.4 [42], which performs hierarchical likelihood ratio tests for phylogenetic congruence, was used to test if the different loci used in this study support alternative topologies (gene-tree/species-tree conflicts; [42]). For this, an alpha level of 0.01 and the WAG substitution model was used with a four-class discretized *Γ* model for rates across sites.

### Gene Tree Reconstruction and Concatenated Phylogenetic Analyses

Posterior distribution of gene trees was reconstructed from each of the individual nuclear data sets and from a data set of the combined nuclear genes using Bayesian Inference analysis (BI) in MrBayes v. 3.1 [43]–[44]. JModeltest v 0.1.1 [45] was used to determine the best nucleotide substitution model using BIC criteria (Table S2). For the concatenated analyses, we used a partitioned approach with model parameters estimated previously. The analyses were initiated from random starting trees, run for 10 million generations with four chains with two independent repetitions (Metropolis-coupled Markov Chain Monte Carlo; [46]) and sampled at intervals of 1000 generations. Default priors were used in all analyses. To evaluate stationarity and convergence between runs, log-likelihood scores were plotted using TRACER v. 1.5 [47]. In addition, we examined the distribution of split frequencies using the online program AWTY [48], in order to assess whether an MCMC analysis has run long enough such that tree topologies were sampled in proportion to their true posterior probability distribution, independent of the apparent stationarity detected in TRACER. Trees generated prior to stationarity were discarded and the rest of the trees were summarized in a majority-rule consensus tree from the four independent runs. For this, the estimated burn-in based on log likelihood plots was 1000 samples (100,000 generations) per chain leaving 9000 samples per chain (18,000 total) for inference. Bayesian posterior probabilities (PP) were assessed at all nodes, and clades with PP≥0.95 were considered well supported [46]. Maximum likelihood (ML) analyses were performed with the program RAxML v. 7.2.8 [49]. Branch support was assessed with 1000 nonparametric bootstrapping replicates using the same model parameters settings as the BI analyses. Final trees were visualized with FigTree v1.3.1 [50].

### Bayesian Concordance Analysis of Gene Trees

Bayesian concordance analysis (BCA) [36] was used to provide an estimate of the level of concordance in reconstructed branches among the posterior distributions of gene trees generated for each nuclear gene. For these analyses, tree files from each single gene obtained in the BI analyses in MrBayes were summarized using the command mbsum included in the BUCKy program [51], with a burn-in of 1000 trees. The BCA was then performed in the program BUCKy v. 1.4.2, with four independent runs and four Markov chain Monte Carlo (MCMC) chains, each with 10 million generations with a burn-in period of 100,000. Five values of the alpha parameter (0.1, 0.5, 1, 5, 10) were tested, which correspond to the prior probability distribution for the number of distinct gene trees [36].

Since we observed in the single gene analyses that loci such as *act* and LSU have poor resolution at low taxonomic ranks, we also tested if their inclusion in the BCA affected the final outcome of the test. For this, two data sets were built: one containing all genes and another containing a subset of the more phylogenetically informative genes (*tub*, *tef1*, ITS, *rpb1*). Default settings were used for all other parameters. A primary concordance tree with clade concordance factors (CF) and their 95% credibility intervals (sample and genome wide) were determined for each one of the two data sets.

### Coalescent-based Species Tree Analysis

We used the program Species Tree Ancestral Reconstruction/Bayesian Evolutionary Analysis by Sampling Trees (*BEAST v1.7.2) [52] to estimate the species tree for the group. The species assignment for each one of the isolates was based on the groupings obtained in the concatenated phylogenetic analyses using BI and ML approaches. All six nuclear gene datasets were used and the nucleotide substitution model was the same used for BI and ML phylogenetic analyses. We repeated each analysis twice and MCMC analyses were run for a total of 30 million generations, sampled trees at intervals of 1000 generations, and a burn-in of 10%. A Yule process was used for the species tree prior; the population size model was set to Piecewise linear and constant root. Default values were used for remaining priors. Convergence was assessed in TRACER v1.5, with the species tree reconstructed after a 25% burn-in using Tree Annotator v1.7.2 [53].

### Polymorphism and Divergence

To better visualize differences within and between clades, we calculated basic nucleotide polymorphism statistics. The program DnaSP v.5 [54] was used to calculate nucleotide diversity (π, average number of nucleotide differences among sequences in a sample; [55]), number of haplotypes (H), and total number of polymorphic sites (N_poly_) within clades, and nucleotide divergence (Dxy, pairwise average number of nucleotide substitutions per site between groups; [56]) between clades. For these calculations, groups to be compared were defined based on clade assignment of each individual in the concatenated ML and Bayesian phylogeny. The randomization test to assess the significance of Dxy values between groups of clades was calculated using 1000 permutations in DnaSP v.5 [54], [57]. Singletons or orphan isolates were not included in these calculations since these methods compare clades of multiple isolates.

## Results

For this study a total of 402 sequences were generated and are available in GenBank (Table S1). The number of variable sites (nucleotides) across the six loci ranged from 0.054 to 0.351. The *rpb1* region showed the highest sequence variability and number of parsimony informative sites, while the ribosomal genes (ITS, LSU) exhibited low levels of variation, approximately more than half that observed in *rpb1* (Table S2). The combined matrix consisted of 4136 aligned nucleotide positions of which 3361 were constant, 159 variable parsimony-uninformative, and 616 variable and parsimony-informative. Sequence alignments for each locus are deposited under doi:10.5061/dryad.q3s66 at the DRYAD data repository (http://datadryad.org/).

### Bayesian and Maximum Likelihood Analyses

**Figure 2:**
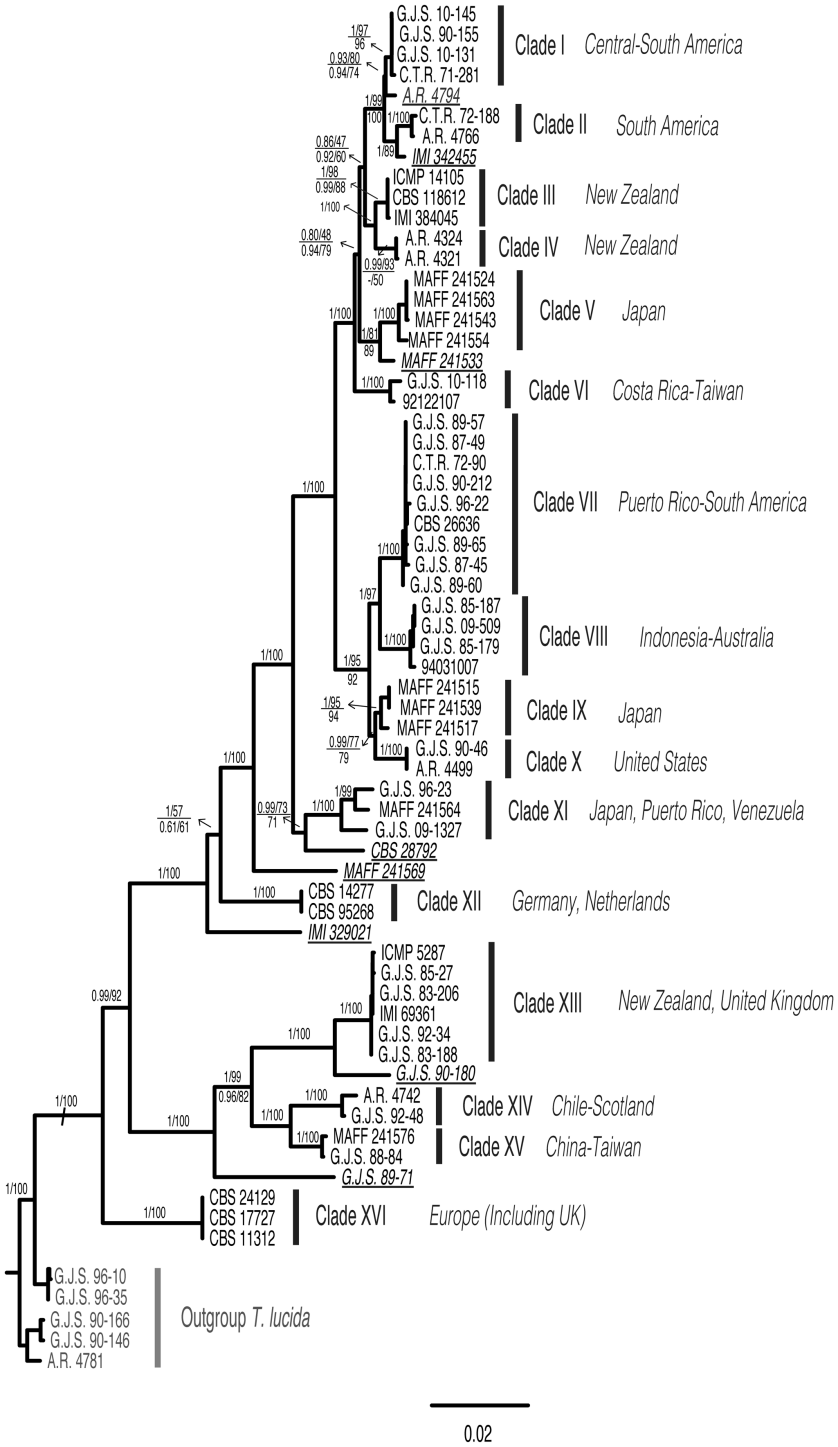
Bayesian phylogram showing relationships among isolates of *T. discophora*-like species based on the concatenated analysis of six loci. Bayesian posterior probabilities and ML bootstrap are indicated above each branch. Bayesian posterior probabilities and ML bootstrap from the concatenated analyses excluding ITS loci are indicated below each branch. No values below branches indicate equal support was found for the different analyses. “−” indicates branch was not recovered/supported.

By combining the evidence found in the concatenated phylogenetic analyses, a total of sixteen putative species were recovered, having significant ML bootstrap (>70%) and BI posterior probability (>0.95) support (Figure 2). Bayesian PP values were usually higher than ML bootstrap support values. The analyses of the concatenated alignment converged quickly to a stationary distribution in the Bayesian analysis run in MrBayes. Effective sample sizes (ESS) of all parameters were at least 300, and most were greater than 1000. The MrBayes majority consensus tree topology shown in Figure 2 was the same as the best ML tree. Significant incongruence was found when the ITS region was included in the concatenated data set. Thus, based on the results obtained by Concaterpillar, ML and MrBayes analyses were also run using a concatenated data set in which ITS was excluded (Figure S1). The branch support obtained when the ITS data set was excluded from the concatenated analysis is also included in Figure 2.

The 16 putative species grouped into three major clusters: the first containing clades I to VI, the second containing clades VII to X, the third containing clades XIII to XV; clades XI and XII appear basal to clades I to X and are surrounded by singletons (single isolate lineages) (Figure 2). Isolates in clade XVI were recovered as the most basal species, being distantly related to the rest of *T. discophora*-like putative species, and close to the outgroup species *T. lucida*. Since *T. discophora* was originally described from Chile, clade XIV constitutes the type locality clade and is thus recognized as true *T. discophora*. Eight isolates were found to form singletons. These singletons or orphan isolates either did not cluster with the closest related putative species having significant branch support, *i.e.,* the branch support decreases if they were included, or they were separated from them by a long branch (Figure 2). The majority of the internal nodes in the phylogeny are resolved and well supported. When the ITS data set was not included in the concatenated analysis, the branch support for the majority of putative species remained significant in both ML and MrBayes analyses, with the exception of clade IV which lost branch support and was resolved as a polytomy (Figure S1). Support for some internal nodes in the ML analysis decreased due to the exclusion of ITS; yet, for the most part, support for internal nodes in MrBayes analyses remained the same (Figure 2).

### Single Gene Tree Analyses

Single gene tree analyses recovered the 16 putative species as observed in the concatenated analyses. In the single gene phylogenies, MrBayes and ML analyses recovered the same clades; however, their positions and those of some singleton isolates differed from that seen in the combined analyses (*rpb1* genealogy shown; Figures S2 and S3). Although some of the clades were not significantly supported, they were also not contradicting, consequently fitting the criteria for species delimitation using genealogical concordance (GCPSR) [19].

According to the analyses carried out in Concaterpillar v.1.4 [42], the inclusion of ITS sequences in the concatenated data set was rejected at P<0.01, indicating its discordance with the rest of the data sets (*act,* LSU, *tef1*, *tub*, *rpb1*). This discordance was restricted to the position of putative species II and XVI (Figures S4 and S5). In the ITS genealogy obtained from MrBayes and ML analyses, the putative species II clustered with the group of putative species IX and it was resolved as a polytomy, in contrast with the rest of the loci where clade II was always associated with the group of putative species I–V. On the other hand, in the MrBayes and ML ITS analyses, clade XVI appeared well supported and basal to clades I–XI. This is contrary to the other single gene topologies and concatenated analyses in which this clade was basal to all clades I–XV. Single gene trees obtained for evolutionarily conserved nuclear loci *act* and LSU showed that relationships among isolates were largely unresolved and resulted in large polytomies (Data not shown).

### Bayesian Concordance Trees and Coalescent-based Species Tree

**Figure 3:**
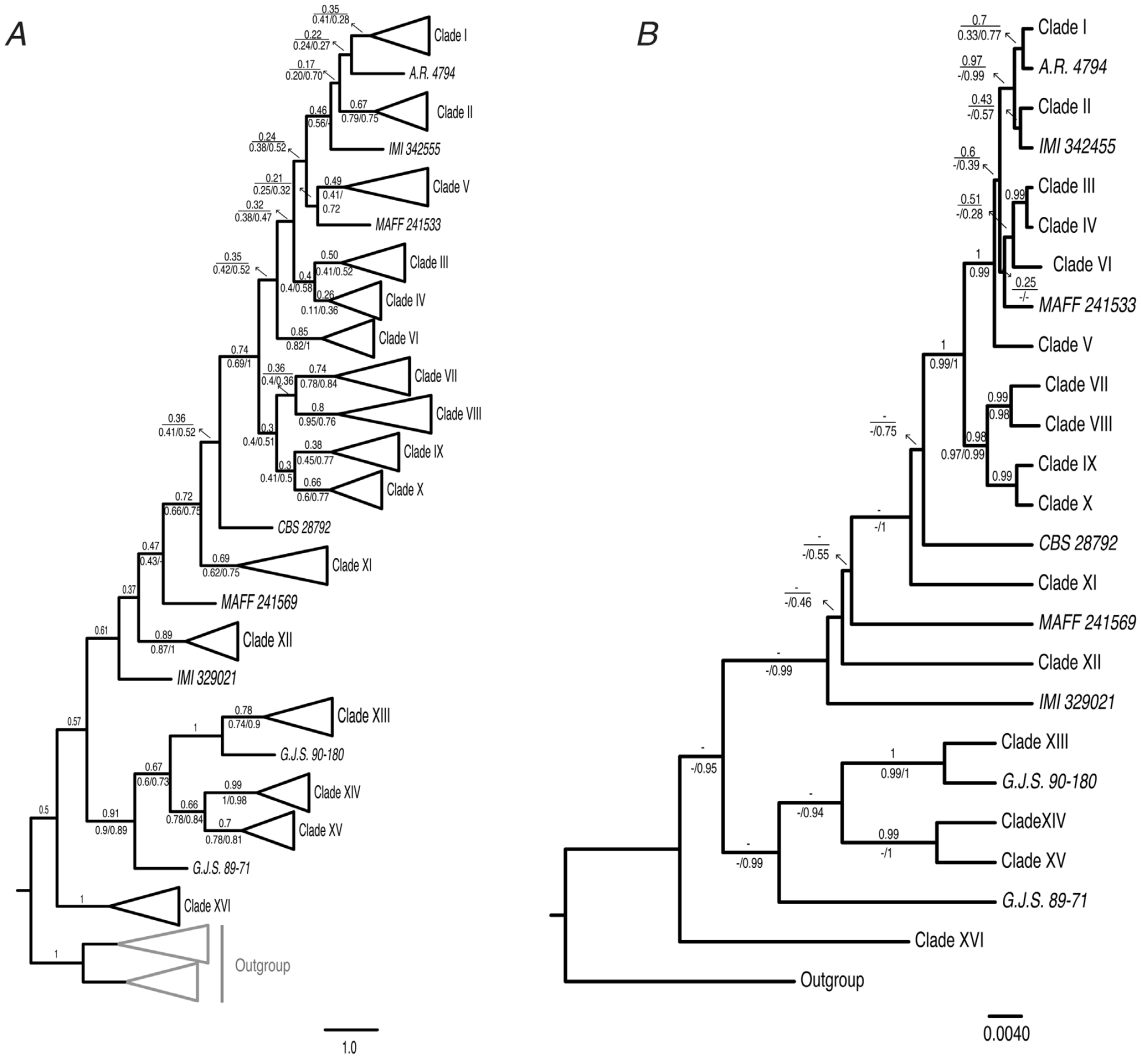
The Bayesian concordance analyses (BCA) tree and coalescent-based species trees. A. Primary concordance tree estimated by BCA; values above branches indicate sample-wide clade concordance factors (CF). Values below branches correspond to sample-wide CF from the analyses of the data set excluding ITS and reduced data set (4-loci), respectively. The primary concordance tree with 95% credibility intervals is included in Figure 5S. B. Maximum clade credibility tree from concatenated analyses in *BEAST. This tree represents the posterior sample with the maximum sum of clade posterior probabilities at the internal nodes. Branch lengths equal to expected substitutions per site. Posterior probabilities of each clade are shown above branches. Values below branches correspond to posterior probabilities obtained in the analyses of the data set excluding ITS and reduced data set (4-loci), respectively. “−” indicates clade not recovered/supported.

Due to the increasing awareness that the sole use of concatenated sequence data for phylogenetic analyses can produce misleading results due to gene tree/species tree discordance [58], we compared the results of standard gene concatenation with those of Bayesian Concordance Analyses (BCA, [36]) and Bayesian Inference of Species Trees [37]. The primary concordance tree obtained from the BCA was similar to the topology obtained in the phylogenetic analyses of the concatenated data set (Figure 3A). However, concordance factor (CF) values for the putative species were much more conservative in this method than the posterior probabilities and bootstrap values obtained in the analyses of the concatenated alignment. CF values for thirteen out of the sixteen putative species were >0.5; while for three clades the CF values were <0.5 (Figure 3A). There was agreement between the calculated sample-wide CF and the extrapolated genome-wide CF, although the genome-wide CF was slightly lower than the sample-wide CF (Figure S6). The groups of putative species XIII-XVI obtained the highest CF values and 95% CI values (sample and genome wide) compared with the rest of clades.

The species tree topology estimated using the coalescent-based method (*BEAST) was somewhat different from that obtained in the concatenated and BCA analyses, even though the branch support for the putative species was significantly high. The main conflict between these two methods was observed in the relationships of putative species V and VI (Figure 3B). The support for internal nodes or the “backbone” of the phylogeny from both BCA and *BEAST was particularly weak (Figures 3A–B). The use of different values for the alpha parameter (the *a priori* degree of incongruence) did not have any effect on the CF of each putative species in the primary concordance tree, given that the method is robust and with the exception of the ITS loci, there are no major conflicts in the data set.

The BCA and *BEAST analyses of all six genes and of the reduced data set excluding *act* and LSU resulted in differences in support values for the putative species. For BCA, concordance factors obtained from the analyses excluding *act* and LSU increased for 11 and decreased for 4 putative species (Figure 3A). Despite the fact that branch support was lower for some putative species, this reduction was less than 20%. On the other hand, the increase in branch support for 11 clades went up to 45.7% (Figure 3A). *BEAST analyses excluding *act* and LSU produced species trees with the same topology as the combined analyses and * BEAST runs with the complete data sets. However, the PP values were generally higher when *act* and LSU were excluded (Figure 3A).

According to the software specifications, *BEAST and BUCKy are designed to identify the underlying species-level phylogeny while accounting for heterogeneity among gene genealogies due to incomplete lineage sorting [36], [59]. The inclusion of a discordant locus data set such ITS would have little or no effect. However, *BEAST and BCA analyses without the ITS dataset resulted in a general decrease in branch support (Figures 3A–B) and differences in tree topology (Figure S7), possibly due to the exclusion of phylogenetically informative sites contained in the ITS region. The support for internal nodes was also low. This is a general trend observed even in the analyses with the complete data set, apparently because the loci used in this study lack sufficient informative sites to resolve deep nodes.

### Genetic Divergence Estimates

The greatest nucleotide diversity (π) within putative species in this complex was recovered from protein coding loci (including exon and intron regions) such as *tef1*, *tub* and *rpb1*, ranging from 0.0005 to 0.0189 (Table 1). In general, all nuclear loci used, either ribosomal or protein coding, showed a high number of unique haplotypes. Many of the putative species showed one single haplotype per nuclear locus. However, clades such as V, VII, VIII, IX, and XI show more than one unique haplotype for the majority of nuclear loci, which also coincides with their high nucleotide diversity. Additionally, genetic distance, as measured by Dxy values obtained between all pairs of putative species, ranged from 0.004 and 0.068 (Table 2). The highest average genetic distances were observed between clusters of putative species 1 (I–XII) and 2 (XIII–XV) with values ranging from 0.031 to 0.067. The putative species containing the pathogenic isolates (clade XVI) is the most distantly related to clusters 1 and 2, with genetic distances ranging from 0.045 to 0.051 (Table 2). The degree of genetic distance between pairs of clades was not related to geographic distance. For example, clades III and XIII contain isolates from New Zealand, which have no clear geographic but genetic boundaries.

**Table 1:** Polymorphism statistic for putative species within the *T. discophora* species-complex.

	*act*		ITS		LSU		*rpb1*		*tef1*		*tub*	
	N/Npoly/h	π	N/Npoly/h	π	N/Npoly/h	π	N/Npoly/h	π	N/Npoly/h	π	N/Npoly/h	π
Clade I	4/0/1	0	4/0/1	0	4/0/1	0	4/0/1	0	4/4/3	0.00239	4/0/1	0
Clade II	2/0/1	0	2/0/1	0	2/0/1	0	2/0/1	0	2/4/2	0.00477	2/0/1	0
Clade III	3/0/1	0	3/0/1	0	3/0/1	0	3/1/2	0.00105	3/0/1	0	3/0/1	0
Clade IV	2/1/2	0.00186	2/0/2	0	2/0/2	0	2/0/1	0	2/0/1	0	2/0/1	0
Clade V	5/3/2	0.00233	5/5/2	0.00404	5/1/2	0.00050	5/12/3 0.00757		5/11/3	0.00645	5/13/3	0.01176
Clade VI	2/2/2	0.00371	2/0/1	0	2/0/1	0	2/3/2 0.00473		2/3/2	0.00359	2/2/2	0.00380
Clade VII	9/2/3	0.00082	9/0/1	0	9/0/1	0	9/0/1	0	9/3/4	0.00079	9/3/3	0.00158
Clade VIII	4/2/2	0.00186	4/0/1	0	4/0/1	0	4/0/1	0	4/2/2	0.00119	4/2/3	0.00222
Clade IX	3/2/2	0.00247	3/0/1	0	3/0/1	0	3/0/1	0	3/11/3	0.00876	3/1/2	0.00127
Clade X	2/0/1	0	2/0/1	0	2/0/1	0	2/0/1	0	2/0/1	0	2/0/1	0
Clade XI	3/4/2	0.00497	3/7/3	0.00970	3/4/3	0.00331	3/17/3	0.01893	3/19/3	0.01580	3/14/2	0.01895
Clade XII	2/0/1	0	2/0/1	0	2/0/1	0	2/0/1	0	2/0/1	0	2/0/1	0
Clade XIII	6/0/1	0	6/0/1	0	6/0/1	0	6/0/1	0	6/2/3	0.00105	6/1/2	0.00064
Clade XIV	2/0/1	0	2/0/1	0	2/0/1	0	2/1/2	0.00160	2/10/2	0.01215	2/0/1	0
Clade XV	2/2/1	0.00372	2/0/1	0	2/0/1	0	2/0/1	0	2/0/1	0	2/2/2	0.00391
Clade XVI	3/0/1	0	3/0/1	0	3/0/1	0	3/0/1	0	3/0/1	0	3/0/1	0
Total	54/43/25	0.01269	54/80/16	0.02597	54/35/13	0.00840	54/197/23	0.0731	54/135/32	0.0315	54/126/26	0.0478

N, number of sequences/individuals, Npoly, number of polymorphic sites, h, number of unique haplotypes, π, nucleotide diversity.

**Table 2:** Nucleotide divergence (Dxy*) for all pairwise comparisons of putative species identified within *T. discophora* species-complex.

	I	II	III	IV	V	VI	VII	VIII	IX	X	XI	XII	XIII	XIV	XV	XVI
Clade I																
Clade II	0.004															
Clade III	0.006	0.008														
Clade IV	0.005	0.007	0.001													
Clade V	0.010	0.011	0.008	0.008												
Clade VI	0.013	0.014	0.010	0.009	0.014											
Clade VII	0.016	0.015	0.013	0.012	0.016	0.016										
Clade VIII	0.018	0.017	0.014	0.014	0.016	0.018	0.009									
Clade IX	0.014	0.013	0.011	0.011	0.014	0.015	0.010	0.010								
Clade X	0.014	0.014	0.013	0.012	0.015	0.016	0.010	0.011	0.006							
Clade XI	0.029	0.029	0.028	0.027	0.029	0.030	0.028	0.028	0.026	0.027						
Clade XII	0.040	0.040	0.038	0.037	0.038	0.040	0.038	0.039	0.037	0.038	0.034					
Clade XIII	0.068	0.069	0.066	0.065	0.066	0.068	0.068	0.067	0.065	0.066	0.068	0.061				
Clade XIV	0.065	0.065	0.063	0.062	0.063	0.065	0.065	0.064	0.063	0.063	0.064	0.058	0.034			
Clade XV	0.062	0.062	0.060	0.059	0.061	0.061	0.061	0.061	0.059	0.060	0.061	0.054	0.031	0.016		
Clade XVI	0.049	0.050	0.048	0.047	0.049	0.050	0.049	0.049	0.048	0.048	0.051	0.044	0.052	0.049	0.045	

* *p*<0.001.

All positions containing gaps were eliminated for a total of 3708 positions. Numbers across the top row correspond to putative species numbers in the first column.

### Ecology and Geographic Distribution of Species

Even though geographical segregation at various levels was observed, it is not a strong character for defining the putative species (Table S1, Figure 2). From the combined phylogenetic analyses we could observe putative species formed by isolates from the same geographic region (Clades III–V, IX, X); isolates from close-by regions (Clades I, VII, VIII, XII); and isolates coming from apparently distant regions (Clades II, VI, XI, XIII–XV, XVI) (Table S1, Figure 2).

Interestingly, a correlation with ecology was observed for all putative species. Isolates assigned to the clades I–XI and XIII–XV were collected in their sexual state, *i.e.,* as fruiting bodies (perithecia) on decaying plant material. Isolates in clade XII were collected as saprobes on soil in the asexual state (‘cylindrocarpon’). Isolates in clade XVI are plant pathogens on several species of *Rubus*, also collected as the sexual state (Table S1; [35]). None of the rest of putative species has been found on *Rubus* species causing disease. Isolates in the outgroup and sister species *T. lucida* were collected as sexual fruiting bodies on decaying plant material. Based on our observations, no host specificity was shown by the putative species. However, the lack of information about the host on which some of the isolates were collected makes it almost impossible to reach a definite conclusion (Table S1).

## Discussion

### Species Delimitation, Diversity and Taxonomy

Utilizing a combination of phylogenetic analyses, genetic distances and species tree inference, we have delineated within the morphospecies *T. discophora* at least 16 distinct putative species. The genetic distances between putative species mostly exceeded standard values of genetic distance (0.01–0.03) used to delimit operational taxonomic units (OTU), revealing them as independent entities. This indicates a >16-fold increase in the species diversity in the genus *Thelonectria* and provides additional evidence for the hyperdiversity of fungi. This high number of putative species is even more surprising considering the relatively few and scattered localities from where we obtained specimens, representing only a tiny portion of their potential geographic distribution. The conclusion that a species is not cosmopolitan has implications for its taxonomy and thus the true diversity of a group of organisms [2], [10], [60]. Revealing the true distribution of a species also has implications in conservation programs, because some of the most important criteria used to assess the conservation status of a species are their geographic distribution, population size and information on how these features are changing over time [61].

Given the lack of diagnostic morphological characters than can be used for taxonomic circumscription within many fungal groups and especially species complexes, the inclusion of other kinds of data such as molecular characters is extremely helpful for establishing species limits [21], [62], [63]. Our species delimitation criterion is the genealogical concordance phylogenetic species recognition (GCPSR) [19], which is based on the convergence of evidence from phylogenetic analyses of unlinked loci, combined with species tree recovery and genetic divergence estimates. The approaches used in this study to reveal phylogenetic relationships among isolates of *T. discophora* uncovered the same relationships and confidently identified 16 previously undetected putative species. For Clades I–II and III–IV, the low values of genetic divergence and short branches may be indicative of their possible recent divergence. The rest of the clades show divergence values higher than 0.01, demonstrating little genetic cohesiveness and independent evolutionary history.

Our phylogenetic analyses also detected eight single isolate lineages or singletons. In systematics, no consensus exists on how singleton lineages should be treated, even though they represent distinctive evolutionary units and make up a considerable portion of the global diversity of species in this group [64]. One possibility could be to include them as part of the closest clade; however, it has been seen that this causes a decrease in the putative species’ support values. Further increase in taxon sampling would determine if they should be separate species and if should be named. Many of these singletons likely constitute rare taxa, which highlight even more the importance of preserving the habitat where they can be found [61].

### Species Tree Reconstruction

Bayesian concordance analyses (BCA) [36] and Bayesian inference of species trees (*BEAST) [37], [52] have been proven as useful complements to the phylogenetic analyses using standard concatenated data as they are able to account for gene tree/species tree discordance. Phylogenies with the concatenated data and BCA estimated the same species relationships, however, different tree topologies were obtained from the *BEAST analyses. The presence of other topologies in *BEAST may indicate that our data set do not contain enough information to accurately infer the species tree by this coalescent method or that different evolutionary histories can affect this method more than others [65]. Additional studies with larger taxon sampling and data sets will allow further analysis of the results of *BEAST compared with BCA and concatenated analyses.

The concatenated phylogeny recovered much higher bootstrap and posterior support values compared to those obtained from the Bayesian Concordance tree estimation (CF values) and coalescent-based species tree strategy (PP values). This is a trend that has been observed in several similar studies [66]–[67]. In the case of CF values in BCA, it is important to note that they are not equivalent to bootstrap percentage or Bayesian posterior probabilities, rather they are estimates of the proportion of sample genes for which a particular clade is true [36], [68]. There is little agreement on what constitutes a significant CF value that would indicate a history of genetic isolation for a particular clade [68]. While we obtained clades with CF values higher than 0.5 (meaning, in an operational way, that the clade is present at least in 50% of the gene genealogies; [68]), clades with low CF values may indicate a difference in the stage of the speciation process [68]. Therefore, we have recovered the dominant phylogenetic history in spite of the low CF recovered for some putative species. The number of genes used in this study did not have a steady effect on CF values. The CF values might depend more on the biology of the organisms (*e.g*., reticulation history), as this knowledge is a major determinant of the sampling effort for taxa and data needed to obtain clades with good CF estimates [67]–[68]. Even though the tree topologies estimated by *BEAST using the reduced data sets, *i.e*., excluding *act* and LSU, and excluding ITS, differed from the concatenated and BCA, the putative species had, in general, high PP support (Figures 3B and S6). This is particularly clear in the topology of the tree obtained with the four-locus data set excluding *act* and LSU, and it is likely to have been strongly influenced by the exclusion of these low-informative data sets. The different parameter models that are used for both reduced data sets can also explain the difference in tree topologies. It has been proven that this method is particularly sensitive to gene discordance and other coalescent processes such as ancestral population sizes [37].

In our study, we found that the ITS gene tree was discordant with the rest of the data sets. This incongruence was not restricted to weak or unresolved nodes as might be expected under a scenario of rapid diversification, evident in the group of putative species I–IV. Rather, this incongruence extends to conflicts involving strongly supported clades, in this case, the putative species XVI. The nodes supporting these clades created the conflict between the ITS locus and the rest of the nuclear data sets analyzed. Putative species XVI includes isolates of *T. discophora* known to be pathogenic to *Rubus* species. This divergence between saprobic and pathogenic species may be recent based on the incongruence found. Based on these results, we could conclude that this incongruence might be due to incomplete lineage sorting, admixture events, or both. Since these two events are often difficult to distinguish with traditional phylogenetic methods as they produce very similar genetic patterns [69], we do not rule out that either process or both are affecting the historical divergence of *T. discophora*-like species.

### Geographic Structure and Cosmopolitanism

Cosmopolitan species are defined as those found all around the world or with a wide geographic distribution. To be truly cosmopolitan, a species needs to have at least the following characteristics: (1) it maintains its genetic cohesiveness mediated by gene flow throughout its distribution [22], and consequently, (2) it possesses highly effective mechanisms for long distance dispersal [2]. In the case of microorganisms, it has been said that microbial taxa will not exhibit endemism because their enormous populations remove dispersal as an effective constraint on geographical range [25]. The results from our research support previous studies with other microorganisms, including fungi, that there are very few truly cosmopolitan species [2], [17], [18]. Because *T. discophora* is a species complex that includes species with restricted geography and ecology, we hypothesize that the limited effective dispersal mechanisms over long geographic distances is affecting their distribution.

The causes and effects of spore dispersal in the geographic distribution of fungi have been poorly studied [9], [70]. Several factors such as spore size, shape, ejection force, and pigmentation can have a significant influence on cosmopolitanism, although fitting these criteria not always implies development of cosmopolitanism [71]. Because of their microscopic size, the forcibly ejected sexual spores of ascomycetous fungi are quickly brought to rest by drag (the force exerted on a body moving in a fluid) [70]. To avoid this, some fungi have evolved minute asexual spores (<5 µm) that are carried easily over long distances by wind currents (*e.g., Aspergillus* spp., *Fusarium oxysporum,* and *Penicillium* spp.) or have spores with drag-minimizing shapes [72]. Other species such as *Ascobolus* spp. and *Sclerotinia sclerotiorum* have the ability to manipulate the local fluid environment surrounding their fruiting bodies to enhance spore dispersal [70]. Finally, dark-pigmented spores protected with melanin against UV radiation and desiccation may survive long-distance dispersion (*e.g*., *Alternaria* spp. and *Sordaria* spp.).

Taking into account the dispersal capabilities and spore morphology in *Thelonectria*, it is unlikely that *T. discophora* has the ability to be a cosmopolitan species. This fungus has asexual spores (macroconidia) that are colorless and longer than 30 µm, sometimes reaching 100 µm; its sexual spores (ascospores) are also colorless or non-melanized (Figure 1). More importantly, both sexual and asexual spores do not have shapes that could improve dispersal, possibly resting after traveling short distances. These characteristics together limit considerably the range of dispersal, and consequently populations experience independent evolutionary trajectories and, ultimately, species divergence. The marked genetic structure observed among the putative species in *T. discophora* likely reflects the interplay between their poor dispersal capabilities and the restrictions to gene flow, either imposed by geographical or reproductive barriers.

The *Thelonectria discophora* complex contains putative species that correlate to geographic origin, meaning one putative species groups isolates from the same or close-by regions. However, this complex also contains putative species that group isolates from apparently distant geographic locations. This phenomenon most probably indicates that members of this species complex show a continuum from regional, indicated by the co-occurrence of putative species with possible niche overlap [73], to large-scale distribution. According to our data, ten putative species were found only in temperate regions such as United States, Europe, Asia and New Zealand. Fewer putative species were found in tropical regions; however, this could be a result of the low taxon sampling that has been made in tropical regions. For example, collections from Venezuela represent at least four putative species. Thus, one may assume that *T. discophora*-like species can be found in tropical and temperate regions equally. Due to their small size and ecological preferences, these fungi have only been collected serendipitously translating into a limited taxon sampling. As it is the case with poorly studied organisms, increasing their collection can further support assumptions about the geographic range of the putative species or about their center of origin. The role of human mediated movement of species of *T. discophora* contributing to the actual geographic distribution of species, cannot be disregarded; however, because these species are not invasive or pathogens of commercial or forest plants, their presence can be overlooked and movement difficult to track.

## Conclusions

The world’s actual magnitude of fungal diversity remains largely under-documented. The continuing destruction and disturbance of natural ecosystems are increasing the chances of massive extinction of species, together with the knowledge about them [10]. Because of their small visible structures, fungi often remain undetected, increasing the potential loss of this group of highly diverse organisms. Under these conditions, efforts to catalogue and explain fungal diversity need to be prioritized treating each diversity group accordingly [74]. A significant percentage of the under-documented diversity of fungi exists as cryptic complexes of species previously recognized as one single species with wide geographical distribution, which have been formally described as separate entities [10], [60]. Based on the evidence presented in this study, the taxonomic status of isolates recognized as *T. discophora* should be reconsidered. This trend is not only found in *Thelonectria*, but also in other genera of the Hypocreales [75]–[76], other ascomycetes [11], [77], and many other microorganisms. At the gross morphological level, *Thelonectria discophora*-like species might appear to be under morphological stasis or an example of many species showing a morphological convergence. However, as for other fungi in the genus *Thelonectria* [78], statistically significant morphological differences could exist. Analyses of populations from a wider geographical range are necessary to untangle the patterns of diversity within this curious species complex, and formal recognition of species in this complex has implications for the diversity of fungi in the family Nectriaceae. Future molecular phylogenetic investigations of geographically and ecologically widespread fungi will most likely uncover far greater levels of biodiversity than currently recognized, as cryptic speciation and regional endemism are revealed.

## Supporting Information

Figure S1 **Maximum likelihood phylogram showing relationships among isolates of** ***T. discophora*** **-like species based on the** ***rpb1*** **loci.** ML bootstrap is indicated on top of each branch. No values below branches indicate branch was not recovered/supported.
(JPG)

Figure S2 **Bayesian phylogram showing relationships among isolates of** ***T. discophora*** **-like species based on the** ***rpb1*** **loci.** Bayesian posterior probabilities are indicated on top of each branch. No values below branches indicate branch was not recovered/supported.
(JPG)

Figure S3 **Maximum likelihood phylogram showing relationships among isolates of** ***T. discophora*** **-like species based on the ITS loci.** ML bootstrap is indicated on top of each branch. No values below branches indicate branch was not recovered/supported.
(JPG)

Figure S4 **Bayesian phylogram showing relationships among isolates of** ***T. discophora*** **-like species based on the ITS loci.** Bayesian posterior probabilities indicated on top of each branch. No values below branches indicate branch was not recovered/supported.
(JPG)

Figure S5 **Primary concordance tree estimated by BCA analysis, values above branches indicate sample-wide 95% CI and below branches indicate genome-wide 95% CI.**
(JPG)

Figure S6 Maximum clade credibility tree from concatenated analyses in *BEAST excluding ITS loci. This tree represents the posterior sample with the maximum sum of clade posterior probabilities at the internal nodes. Branch lengths equal to expected substitutions per site in concatenated data set. Posterior probabilities of each clade are shown above branches.
(JPG)

Figure S7 **Bayesian phylogram showing relationships among isolates of** ***T. discophora*** **-like species when excluding ITS loci.** This tree represents the posterior sample with the maximum sum of clade posterior probabilities at the internal nodes. Branch lengths equal to expected substitutions per site in concatenated data set. ML bootstrap support are shown above branches. BI posterior probabilities are shown below branches. No values above or below branches indicate branch was not recovered/supported.
(JPG)

Table S1 **Taxa used in this study, including information about the origin of the fungal material, collection codes and GenBank accession numbers.**
(DOCX)

Table S2 **Detailed information about specimens used in this study.**
(DOCX)

Table S3 **List of molecular markers and descriptive statistics for the six loci used in this study.**
(DOCX)
