# Supplementary figures and images for: Not as Ubiquitous as We Thought: Taxonomic Crypsis, Hidden Diversity and Cryptic Speciation in the Cosmopolitan Fungus Thelonectria discophora (Nectriaceae, Hypocreales, Ascomycota)
Source: PLoS One. 2013 Oct 18;8(10):e76737. doi: 10.1371/journal.pone.0076737 (PMC3799981; doi:10.1371/journal.pone.0076737)

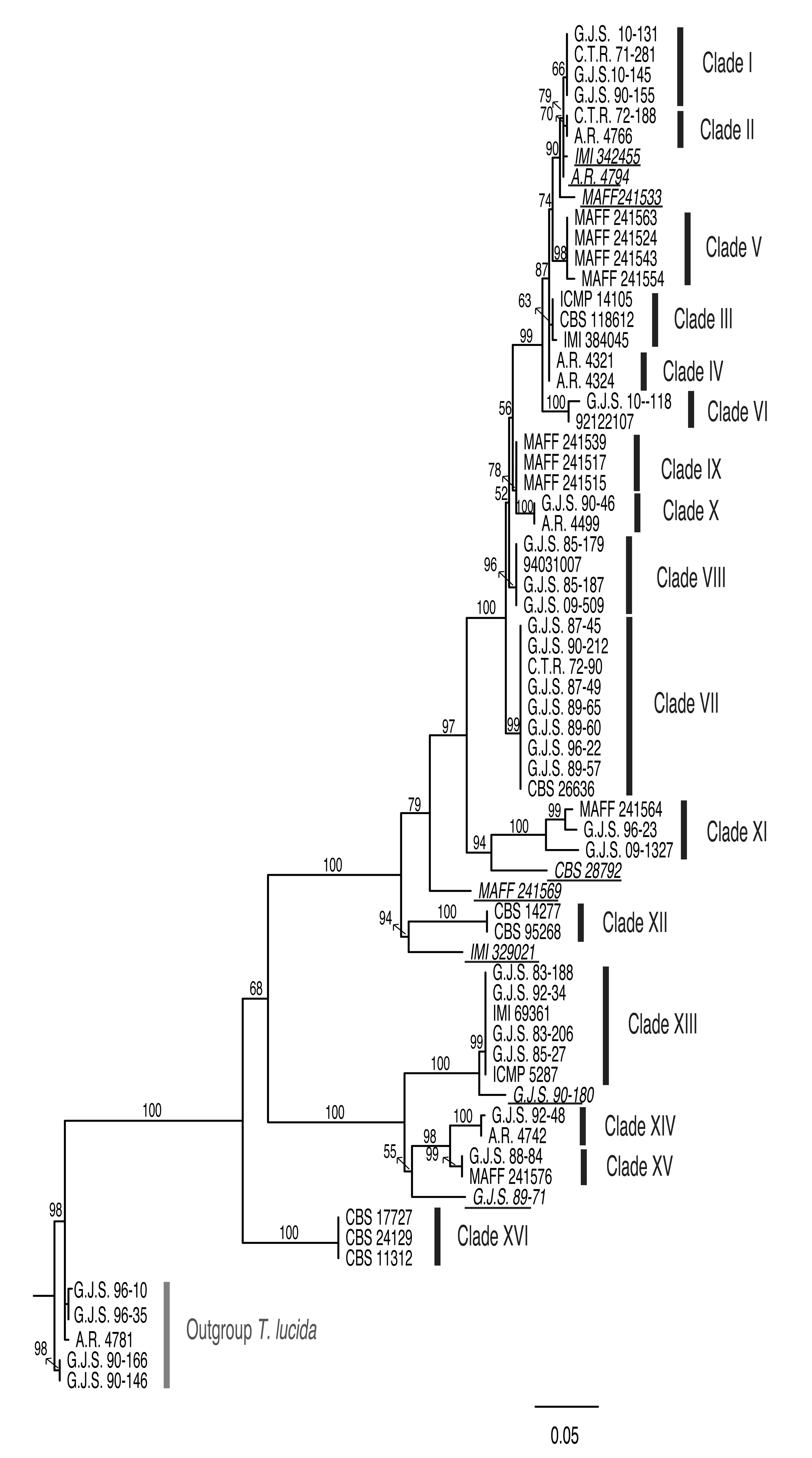

Supplement: Figure S1 — Maximum likelihood phylogram showing relationships among isolates of T. discophora -like species based on the rpb1 loci. ML bootstrap is indicated on top of each branch. No values below branches indicate branch was not recovered/supported. (JPG) [file pone.0076737.s001.jpg]

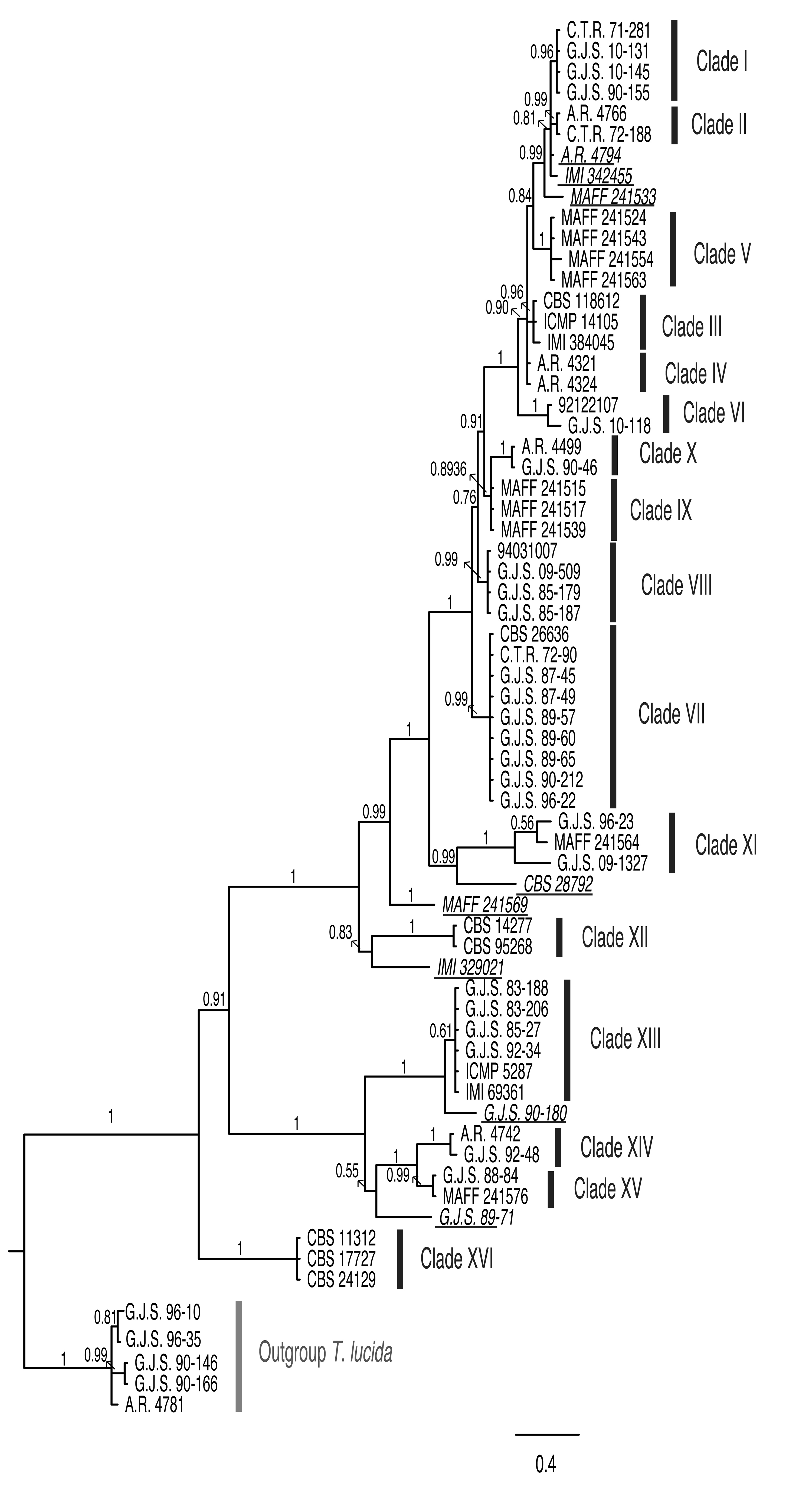

Supplement: Figure S2 — Bayesian phylogram showing relationships among isolates of T. discophora -like species based on the rpb1 loci. Bayesian posterior probabilities are indicated on top of each branch. No values below branches indicate branch was not recovered/supported. (JPG) [file pone.0076737.s002.jpg]

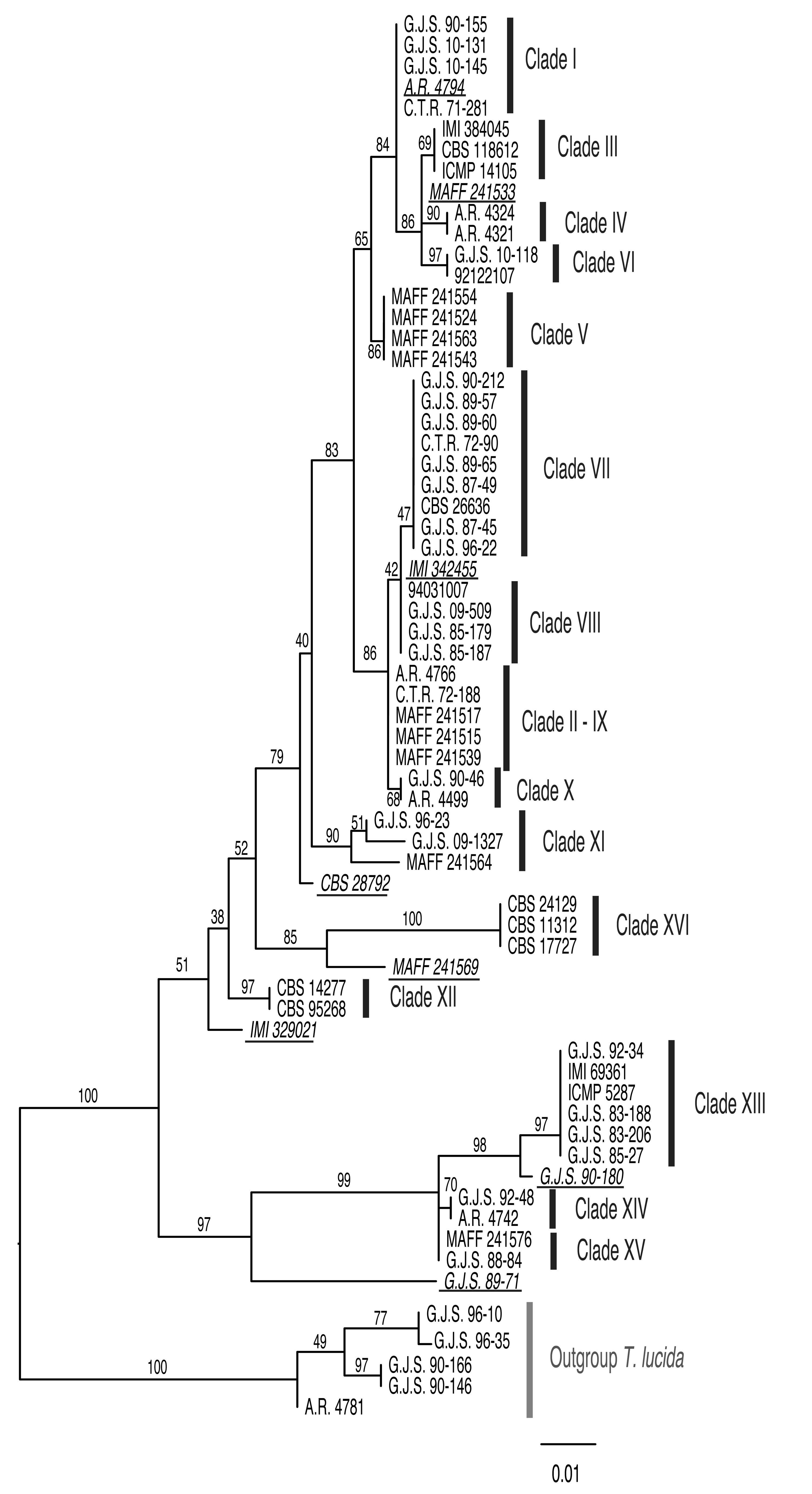

Supplement: Figure S3 — Maximum likelihood phylogram showing relationships among isolates of T. discophora -like species based on the ITS loci. ML bootstrap is indicated on top of each branch. No values below branches indicate branch was not recovered/supported. (JPG) [file pone.0076737.s003.jpg]

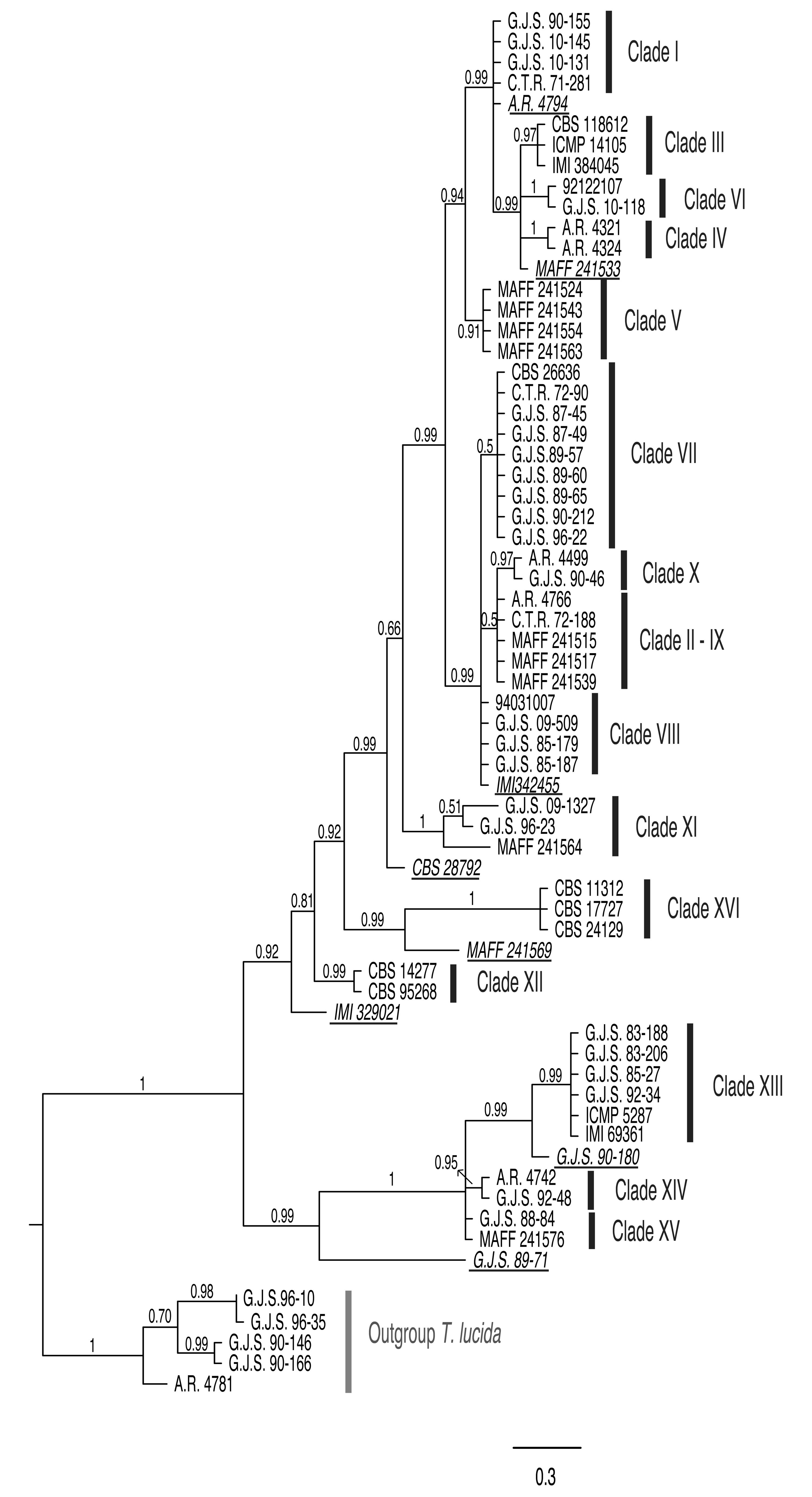

Supplement: Figure S4 — Bayesian phylogram showing relationships among isolates of T. discophora -like species based on the ITS loci. Bayesian posterior probabilities indicated on top of each branch. No values below branches indicate branch was not recovered/supported. (JPG) [file pone.0076737.s004.jpg]

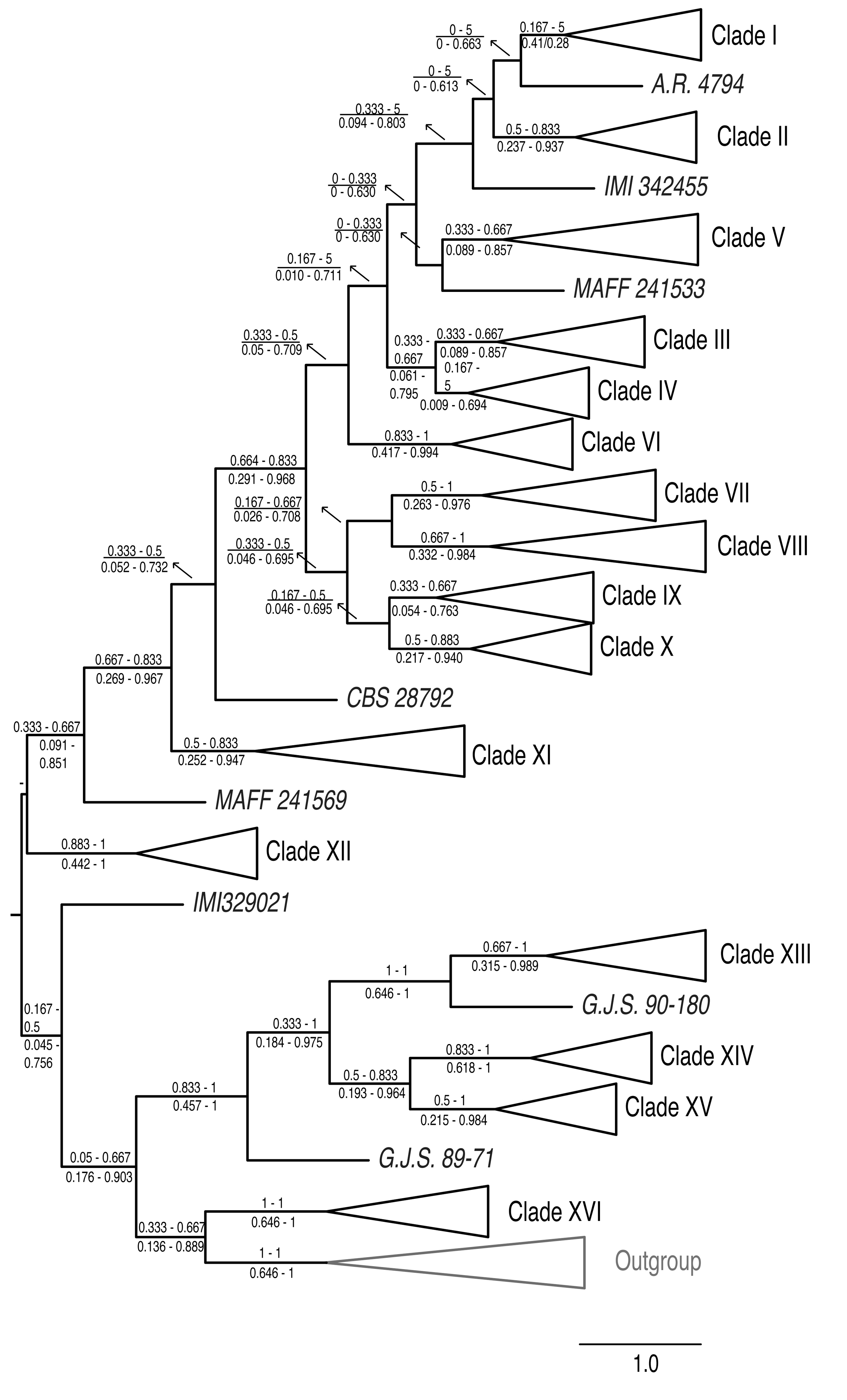

Supplement: Figure S5 — Primary concordance tree estimated by BCA analysis, values above branches indicate sample-wide 95% CI and below branches indicate genome-wide 95% CI. (JPG) [file pone.0076737.s005.jpg]

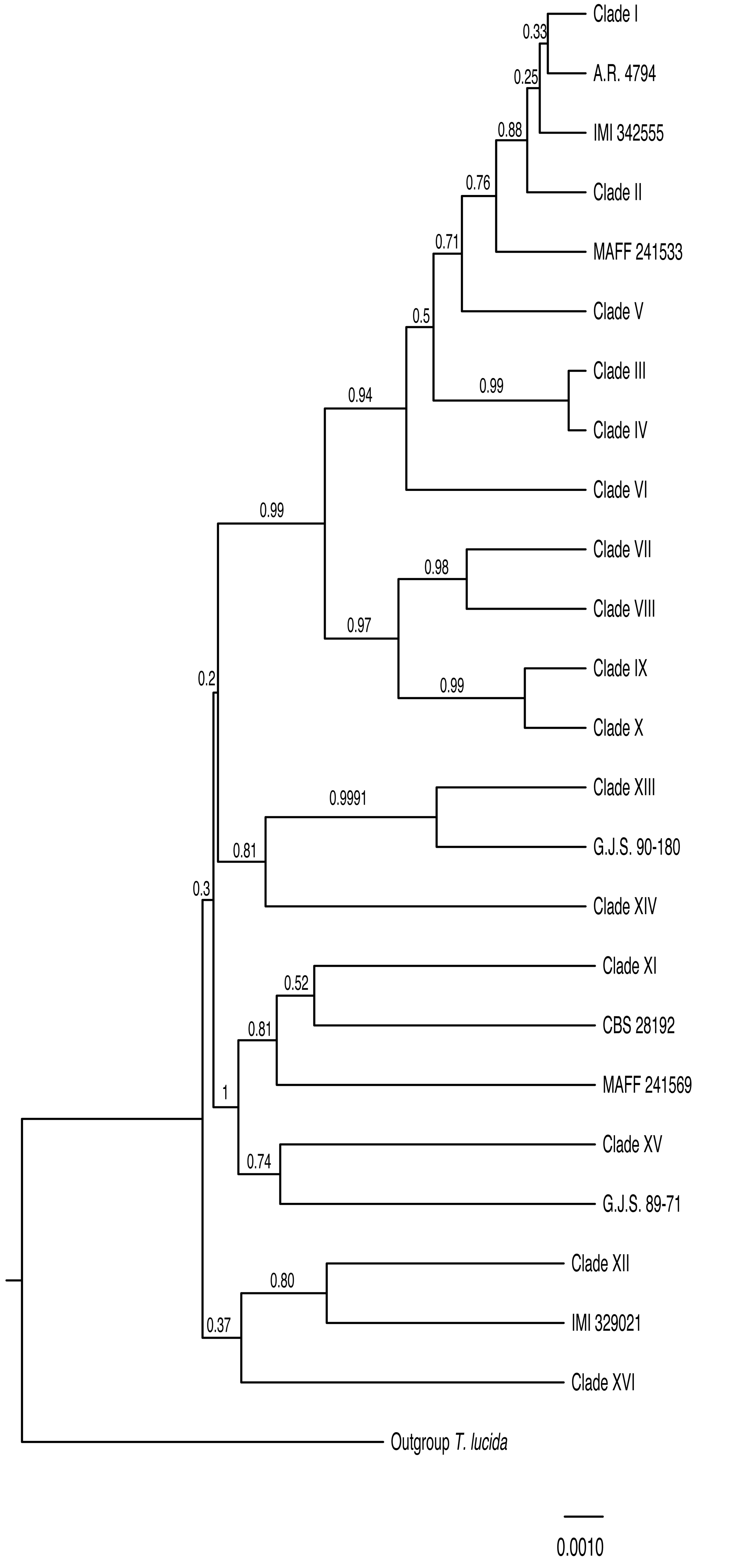

Supplement: Figure S6 — Maximum clade credibility tree from concatenated analyses in *BEAST excluding ITS loci. This tree represents the posterior sample with the maximum sum of clade posterior probabilities at the internal nodes. Branch lengths equal to expected substitutions per site in concatenated data set. Posterior probabilities of each clade are shown above branches. (JPG) [file pone.0076737.s006.jpg]

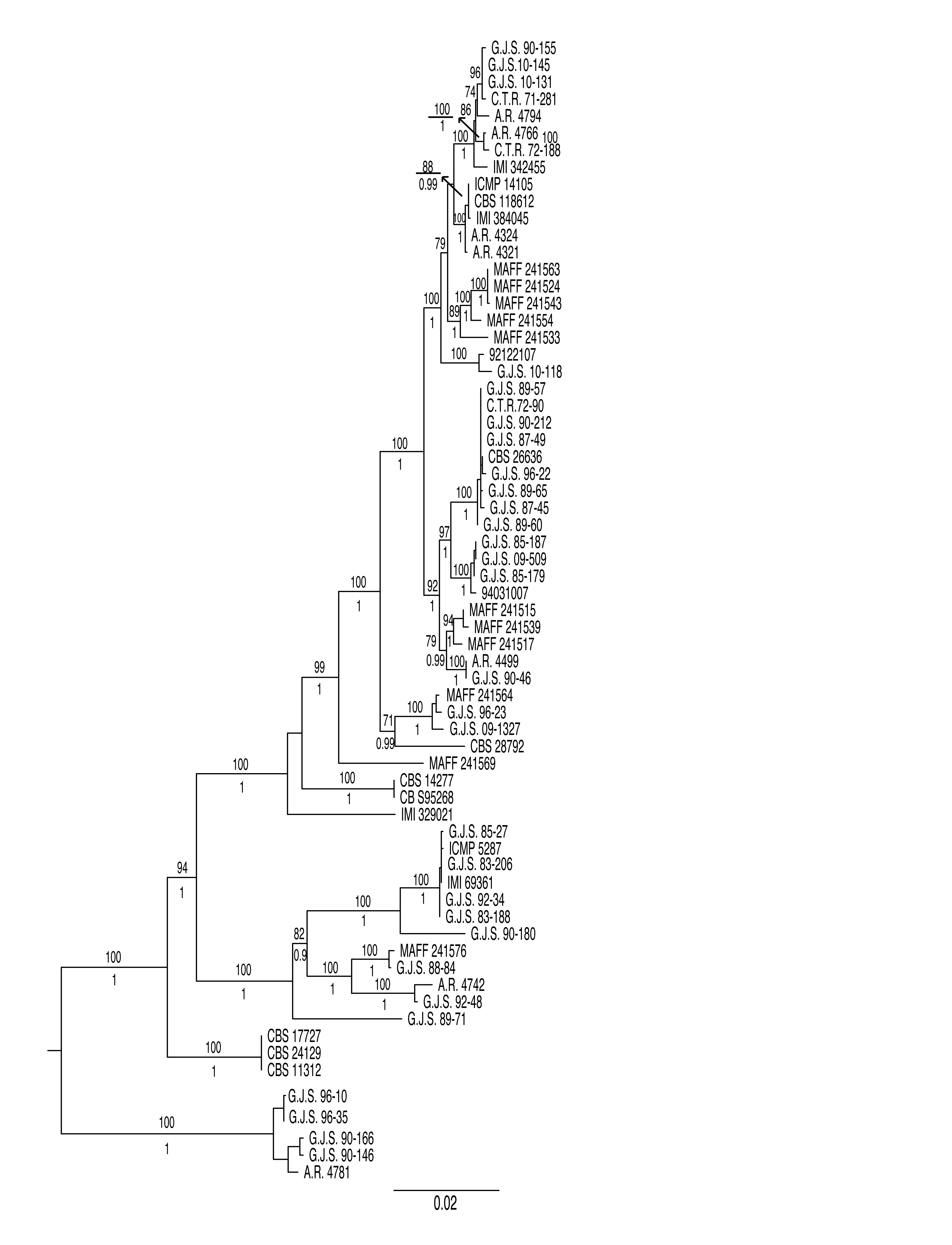

Supplement: Figure S7 — Bayesian phylogram showing relationships among isolates of T. discophora -like species when excluding ITS loci. This tree represents the posterior sample with the maximum sum of clade posterior probabilities at the internal nodes. Branch lengths equal to expected substitutions per site in concatenated data set. ML bootstrap support are shown above branches. BI posterior probabilities are shown below branches. No values above or below branches indicate branch was not recovered/supported. (JPG) [file pone.0076737.s007.jpg]
