# Supplementary material for: Not as Ubiquitous as We Thought: Taxonomic Crypsis, Hidden Diversity and Cryptic Speciation in the Cosmopolitan Fungus Thelonectria discophora (Nectriaceae, Hypocreales, Ascomycota)
Source: PLoS One. 2013 Oct 18;8(10):e76737. doi: 10.1371/journal.pone.0076737 (PMC3799981; doi:10.1371/journal.pone.0076737)
Supplement: Table S1 — Taxa used in this study, including information about the origin of the fungal material, collection codes and GenBank accession numbers. (DOCX) [file pone.0076737.s008.docx]

Table S1. Taxa used in this study, including information about the origin of the fungal material, collection codes and

GenBank accession numbers

| Strain | Code | Host | Origin | GenBank accession numbers | | | | | |
| --- | --- | --- | --- | --- | --- | --- | --- | --- | --- |
|  |  |  |  | *act* | ITS | LSU | *rpb1* | *tef1* | *tub* |
| *Cyl. ianthothele* | CBS 118612 | *Quercus rubur* | New Zealand | KC121381 | KC153719 | KC121445 | KC153912 | KC153848 | KC153784 |
| *Cyl. ianthothele var. majus* | CBS 28792 | On soil | Brazil | KC121386 | KC153724 | KC121450 | KC153917 | KC153853 | KC153789 |
| *Cyl. ianthothele var. majus* | CBS 95268 | On soil | Germany | KC121387 | KC153725 | KC121451 | KC153918 | KC153854 | KC153790 |
| *Neo. discophora var. rubi* | CBS 11312 | *Rubus idaeus* | Switzerland | KC121380 | KC153718 | KC121444 | KC153911 | KC153847 | KC153783 |
| *Neo. discophora var. rubi* | CBS 14277 | On soil | Netherlands | KC121382 | KC153720 | KC121446 | KC153913 | KC153849 | KC153785 |
| *Neo. discophora var. rubi* | CBS 17727 | *Rubus idaeus* | England | KC121383 | KC153721 | KC121447 | KC153914 | KC153850 | KC153786 |
| *Neo. discophora var. rubi* | CBS 24129 | *Rubus idaeus* | Scotland | KC121384 | KC153722 | KC121448 | KC153915 | KC153851 | KC153787 |
| *Neo. discophora var. rubi* | ICMP 14105 | Unknown | New Zealand | KC121420 | KC153758 | KC121484 | KC153951 | KC153887 | KC153823 |
| *T. discophora* | 92122107  (=CBS 134038) | unknown | Taiwan | KC121373 | KC153711 | KC121437 | KC153904 | KC153840 | KC153775 |
| *T. discophora* | 94031007  (=CBS 134039) | unknown | Taiwan | KC121374 | KC153712 | KC121438 | KC153905 | KC153841 | KC153776 |
| *T. discophora* | A.R. 4321  (=CBS 134033) | *Pinus radiata* | New Zealand | KC121375 | KC153713 | KC121439 | KC153906 | KC153842 | KC153777 |
| *T. discophora* | A.R. 4324  (=CBS 125153) | *Pinus radiata* | New Zealand | HM352875 | HM364294 | HM364307 | HM364326 | HM364345 | HM352860 |
| *T. discophora* | A.R. 4499  (=CBS 125172) | *Fagus grandifolia* | U.S | HM352877 | HM364296 | HM364309 | HM364327 | HM364347 | HM364327 |
| *T. discophora* | A.R 4742  (=CBS 134034) | *Tepualia stipularis* | Chile | KC121376 | KC153714 | KC121440 | KC153907 | KC153843 | KC153779 |
| *T. discophora* | A.R. 4766  (=CBS 134035) | Unknown | Argentina | KC121377 | KC153715 | KC121441 | KC153908 | KC153844 | KC153780 |
| *T. discophora* | A.R. 4794  (=CBS 134037) | Unknown | Argentina | KC121379 | KC153717 | KC121443 | KC153910 | KC153846 | KC153782 |
| *T. discophora* | CBS 26636 | unknown | Germany | KC121385 | KC153723 | KC121449 | KC153916 | KC153852 | KC153788 |
| *T. discophora* | C.T.R. 71-281 (=CBS 112458) | unknown | Venezuela | KC121388 | KC153726 | KC121452 | KC153919 | KC153855 | KC153791 |
| *T. discophora* | C.T.R. 72-188  (=CBS 134040) | Unknown | Venezuela | KC121389 | KC153727 | KC121453 | KC153920 | KC153856 | KC153792 |
| *T. discophora* | C.T.R. 72-90 | Unknown palm | Venezuela | KC121390 | KC153728 | KC121454 | KC153921 | KC153857 | KC153793 |
| *T. discophora* | G.J.S. 09-1327  (=CBS 134022) | Unknown | Venezuela | KC121391 | KC153729 | KC121455 | KC153922 | KC153858 | KC153794 |
| *T. discophora* | G.J.S. 09-509 | *Acacia celsa* | Australia | KC121392 | KC153730 | KC121456 | KC153923 | KC153859 | KC153795 |
| *T. discophora* | G.J.S. 10-118  (=CBS 134023) | Unknown | Costa Rica | KC121393 | KC153731 | KC121457 | KC153924 | KC153860 | KC153796 |
| *T. discophora* | G.J.S. 10-131  (=CBS 134024) | Unknown | Costa Rica | KC121394 | KC153732 | KC121458 | KC153925 | KC153861 | KC153797 |
| *T. discophora* | G.J.S. 10-145  (=CBS 134025) | Unknown | Costa Rica | KC121395 | KC153733 | KC121459 | KC153926 | KC153862 | KC153798 |
| *T. discophora* | G.J.S. 83-188  (=IMI 326256) | *Fuchsia exorticata* | New Zealand | KC121396 | KC153734 | KC121460 | KC153927 | KC153863 | KC153799 |
| *T. discophora* | G.J.S. 83-206  (=IMI 326258) | Unknown | New Zealand | KC121397 | KC153735 | KC121461 | KC153928 | KC153864 | KC153800 |
| *T. discophora* | G.J.S. 85-179  (=IMI 329113) | Unknown | Indonesia | KC121398 | KC153736 | KC121462 | KC153929 | KC153865 | KC153801 |
| *T. discophora* | G.J.S. 85-187 (=ATCC 76478) | Unknown | Indonesia | KC121399 | KC153737 | KC121463 | KC153930 | KC153866 | KC153802 |
| *T. discophora* | G.J.S. 85-27  (=CBS 112457) | unknown | New Zealand | KC121400 | KC153738 | KC121464 | KC153931 | KC153867 | KC153803 |
| *T. discophora* | G.J.S. 87-45  (=IMI 325855) | unknown | Guyana | KC121401 | KC153739 | KC121465 | KC153932 | KC153868 | KC153804 |
| *T. discophora* | G.J.S. 87-49 (=CBS 112461) | Unknown | Guyana | KC121402 | KC153740 | KC121466 | KC153933 | KC153869 | KC153805 |
| *T. discophora* | G.J.S. 88-84  (=IMI 348190) | Unknown | China | KC121403 | KC153741 | KC121467 | KC153934 | KC153870 | KC153806 |
| *T. discophora* | G.J.S. 89-57 (=CBS 112459) | Unknown | Guyana | KC121404 | KC153742 | KC121468 | KC153935 | KC153871 | KC153807 |
| *T. discophora* | G.J.S. 89-60 | Unknown | Guyana | KC121405 | KC153743 | KC121469 | KC153936 | KC153872 | KC153808 |
| *T. discophora* | G.J.S. 89-65 (=CBS 123970) | Unknown | Guyana | KC121406 | KC153744 | KC121470 | KC153937 | KC153873 | KC153809 |
| *T. discophora* | G.J.S. 89-71  (= CBS 134026) | Unknown | Guyana | KC121407 | KC153745 | KC121471 | KC153938 | KC153874 | KC153810 |
| *T. discophora* | G.J.S. 90-155 (=CBS 123966) | Unknown palm | Venezuela | KC121409 | KC153747 | KC121473 | KC153940 | KC153876 | KC153812 |
| *T. discophora* | G.J.S. 90-180  (=CBS 134027) | Unknown | Venezuela | KC121411 | KC153749 | KC121475 | KC153942 | KC153878 | KC153814 |
| *T. discophora* | G.J.S. 90-212  (=CBS 134028) | Unknown | Venezuela | KC121412 | KC153750 | KC121476 | KC153943 | KC153879 | KC153815 |
| *T. discophora* | G.J.S. 90-46  (=CBS 134029) | *Quercus* sp. | U.S | KC121413 | KC153751 | KC121477 | KC153944 | KC153880 | KC153816 |
| *T. discophora* | G.J.S. 92-34  (=CBS 134030) | Unknown | Scotland | KC121414 | KC153752 | KC121478 | KC153945 | KC153881 | KC153817 |
| *T. discophora* | G.J.S. 92-48  (=CBS 134031) | *Aesculus* sp. | Scotland | KC121415 | KC153753 | KC121479 | KC153946 | KC153882 | KC153818 |
| *T. discophora* | G.J.S 96-22  (=IMI 370946) | *Ocoter* sp. | Puerto Rico | KC121417 | KC153755 | KC121481 | KC153948 | KC153884 | KC153820 |
| *T. discophora* | G.J.S. 96-23 (=IMI 370947) | Unknown | Puerto Rico | KC121418 | KC153756 | KC121482 | KC153949 | KC153885 | KC153821 |
| *T. discophora* | ICMP 5287 | Unknown | New Zealand | KC121421 | KC153759 | KC121485 | KC153952 | KC153888 | KC153824 |
| *T. discophora* | IMI 329021 | [*Beilschmiedia tawa*](http://cabi.bio-aware.com/BioloMICS.aspx?Link=T&TableKey=1373880000000010&Rec=436&Fields=All) | New Zealand | KC121422 | KC153760 | KC121486 | KC153953 | KC153889 | KC153825 |
| *T. discophora* | IMI 342455 | Unknown | Kenya | KC121423 | KC153761 | KC121487 | KC153954 | KC153890 | KC153826 |
| *T. discophora* | IMI 384045 | Unknown | New Zealand | KC121424 | KC153762 | KC121488 | KC153955 | KC153891 | KC153827 |
| *T. discophora* | IMI 69361 | *Smyrnium olusatrum* | UK | KC121425 | KC153763 | KC121489 | KC153956 | KC153892 | KC153828 |
| *T. discophora* | MAFF 241515 | Unknown | Japan | KC121426 | KC153764 | KC121490 | KC153957 | KC153893 | KC153829 |
| *T. discophora* | MAFF 241517 | *Cryptomeria japonica* | Japan | KC121427 | KC153765 | KC121491 | KC153958 | KC153894 | KC153830 |
| *T. discophora* | MAFF 241524 | Unknown | Japan | KC121428 | KC153766 | KC121492 | KC153959 | KC153895 | KC153831 |
| *T. discophora* | MAFF 241533 | Unknown | Japan | KC121429 | KC153767 | KC121493 | KC153960 | KC153896 | KC153832 |
| *T. discophora* | MAFF 241539 | Unknown | Japan | KC121430 | KC153768 | KC121494 | KC153961 | KC153897 | KC153833 |
| *T. discophora* | MAFF 241543 | Unknown | Japan | KC121431 | KC153769 | KC121495 | KC153962 | KC153898 | KC153834 |
| *T. discophora* | MAFF 241554 | Unknown | Japan | KC121432 | KC153770 | KC121496 | KC153963 | KC153899 | KC153835 |
| *T. discophora* | MAFF241563 | *Fagus crenata* | Japan | KC121433 | KC153771 | KC121497 | KC153964 | KC153900 | KC153836 |
| *T. discophora* | MAFF 241564 | Unknown | Japan | KC121434 | KC153772 | KC121498 | KC153965 | KC153901 | KC153837 |
| *T. discophora* | MAFF 241569 | Unknown | Japan | KC121435 | KC153773 | KC121499 | KC153966 | KC153902 | KC153838 |
| *T. discophora* | MAFF 241576 | Unknown | Japan | KC121436 | KC153774 | KC121500 | KC153967 | KC153903 | KC153839 |
| *T. lucida* | A.R 4781  (=CBS 134036) | Unknown | Argentina | KC121378 | KC153716 | KC121442 | KC153909 | KC153845 | KC153781 |
| *T. lucida* | G.J.S 90-146  (=CBS 134032) | Unknown | Venezuela | KC121408 | KC153746 | KC121472 | KC153939 | KC153875 | KC153811 |
| *T. lucida* | G.J.S 90-166  (=CBS 126099) | Unknown | Venezuela | KC121410 | KC153748 | KC121474 | KC153941 | KC153877 | KC153813 |
| *T. lucida* | G.J.S 96-10  (=IMI 370944) | Unknown | Puerto Rico | KC121416 | KC153754 | KC121480 | KC153947 | KC153883 | KC153819 |
| *T. lucida* | G.J.S 96-35  (=CBS 112456) | Unknown | Puerto Rico | KC121419 | KC153757 | KC121483 | KC153950 | KC153886 | KC153822 |
