# Supplementary material for: Not as Ubiquitous as We Thought: Taxonomic Crypsis, Hidden Diversity and Cryptic Speciation in the Cosmopolitan Fungus Thelonectria discophora (Nectriaceae, Hypocreales, Ascomycota)
Source: PLoS One. 2013 Oct 18;8(10):e76737. doi: 10.1371/journal.pone.0076737 (PMC3799981; doi:10.1371/journal.pone.0076737)
Supplement: Table S2 — Detailed information about specimens used in this study. (DOCX) [file pone.0076737.s009.docx]

| Table S2. Detailed information about specimens used in this study. |
| --- |
|  |
| COSTA RICA. Heredia Province, Braulio Carrillo National Park, Zurqui Street entrance, 10°02’N 84°01’W, 1562 m, on bark, 13 March 2010, C. Salgado, C. Herrera, Y. Hirooka, A. Rossman, G.J. Samuels, P. Chaverri PC1001 (BPI, culture G.J.S. 10-118 = CBS 134023). |
|  |
| COSTA RICA. Heredia Province, Braulio Carrillo National Park, Zurqui Street entrance, 10°03’N 84°01’W, 1734 m, on bark of undetermined twigs, 14 March 2010, C. Salgado, C. Herrera, Y. Hirooka, A. Rossman, G.J. Samuels, P. Chaverri PC1060 (BPI, culture G.J.S. 10-131 = CBS 134024). |
|  |
| COSTA RICA. Heredia Province, Braulio Carrillo National Park, Zurqui Street entrance, 10°03’N 84°01’W, 1734 m, on bark of undetermined dead tree, 14 March 2010, C. Salgado, C. Herrera, Y. Hirooka, A. Rossman, G.J. Samuels, P. Chaverri PC1081 (BPI, culture G.J.S. 10-145 = CBS 134025). |
|  |
| CHILE. Llanquihue Province: Los Lagos Region, Vicente Perez Rosales National Park, on wood of a recently killed *Tepualia stipularis* tree, April 2011, Andrés de Errasti (BPI, culture A.R. 4742 = CBS 134034). |
|  |
| SCOTLAND. Cowal Peninsula, Argyll Forest Park, ca. 10 km north of Dunoon, Yourger Botanic Garden 50–100 m, on *Aesculus* sp. dead branchlets, 11-13 April 1992, G.J. Samuels, D. Brayford, (BPI 802901, culture G.J.S. 92-48 = CBS 134031). |
|  |
| SCOTLAND. Cowal Peninsula, Argyll Forest Park, ca 5 km south of Strachur along river Cur, vic Glenbranter Village, Lauder Broadleaf Walk ca 50 m, on bark of unidentified dead hardwood tree, 12 Apr 1992, G.J. Samuels, D. Brayford (BPI 802649, culture G.J.S. 92-34 = CBS 134030). |
|  |
| ENGLAND. Norfolk County: North Wootton, on bark of unknown plant, Jan 1897, C.B. Plowright (RBGE E00456070). Surlingham City, on *Smyrnium olusatrum* seeds, 1957, E.A. Ellis (ex epitype culture IMI 69361). |
|  |
| JAPAN. Nagano Prefecture, Sugadaira, Ueda City, on twigs, 02 Sept 2006, Y. Hirooka (TPP-h548, BPI 881963, culture MAFF 241576). |
|  |
| CHINA. Yunnan Province, Lijiang region, on bark submerged in stream. G.J. Samuels (only culture examined G.J.S. 88-84 = IMI 348190). |
|  |
| GERMANY. Kiel-Kitzeberg, on wheat field soil, Dec 1968, W. Gams (Extype culture CBS 95268). |
|  |
| NETHERLANDS. Wageningen region, on roots on clay soil, Jan 1977, J.W. Veenbaas-Rijks (culture CBS 14277). |
|  |
| NEW ZEALAND. Auckland, on *Quercus rubur*, March 2005, C.F. Hill (culture CBS 118612). |
|  |
| NEW ZEALAND. Bay of Plenty, Tauranga Locality, on rot root of unknown proteaceus plant, April 1 2000, C.F. Hill (culture ICMP 14105). On root of unknown dead plant, Jul 2000 (culture IMI 384045). |
|  |
| NEW ZEALAND. Southland, Catlin’s State Forest Park, Lake Wilkie, on bark of unidentified tree, 18 Apr 1985, G.J. Samuels, P.K. Buchanan, L.M. Kohn (PDD 50050, BPI 802469, culture G.J.S. 85-27 = CBS 112457). |
| NEW ZEALAND. South Island, Westland, Franz Joseph, track to Lake Wombat, on bark of Fuchsia excorticata, 10 Apr 1983, G.J. Samuels, R.H. Petersen (PDD 46365, BPI 1109329, culture G.J.S. 83-188 = IMI 326256). |
|  |
| NEW ZEALAND. Waitomo, on bark of indetermined tree, 26 Apr 1983, G.J. Samuels, P.R. Johnston, R.H. Petersen (PDD 46410, culture G.J.S. 83-206 = IMI 326258); on *Pinus radiata*, 01 Nov 1965, J. M. Dingley (culture ICMP 5287). SWITZERLAND. May 1981, O. Petrini (culture CBS 32881). |
|  |
| NEW ZEALAND. Bay of Plenty: Rotorua, Whakarewarewa, on bark of Pinus radiata, Sept 1949, G.B. Rawlings (PDD 7510, ex-type culture (epitypified, from Rotura in Bay of Plenty, same place of type locality) A.R. 4324 = CBS 125153)). |
|  |
| NEW ZEALAND. Northland: on *Pinus radiata*, 10 Sep 2003, (culture only, A.R. 4321 = CBS 134033). |
|  |
| GUYANA. Cuyuni-Marazuni: Mazaruni Subregion, VII-2, along Koatse river, ca. 2 km east of pong River, ca hr walk west of Chinoweing, 05°28’N 60°04’W, 600-650 m, Feb-March 1987, on wood, G.J. Samuels, J. Pipoly, G. Gharbarran, J. Chin, R. Edwards (BPI 747133, culture G.J.S. 87-45 = IMI325855). |
|  |
| GUYANA. Cuyuni-Marazuni: Mazaruni Subregion, VII-2, along Koatse river, ca. 2 km east of pong River, ca hr walk west of Chinoweing, 05°28’N 60°04’W, 600-650 m, 28 Feb 1987, on dead branchlets of recently dead tree, G.J. Samuels, J. Pipoly, G. Gharbarran, J. Chin, R. Edwards (BPI 744725, culture G.J.S. 87-49 = CBS 112461); |
|  |
| GUYANA. Potaro-Sinupari Region, base of Mt. Wokomung, ca 5.5 hr walk NE of Kopinang Village in legume dominated forest, 05°05’N 59°49’W, 27 Jun 1989,on bark of recently fallen tree, G.J. Samuels, B.M. Boom, G. Bacchus (NYBG 6269A, culture G.J.S. 89-57 = CBS 112459). |
|  |
| GUYANA. 720 m, G.J. Samuels, B.M. Boom, G. Bacchus (NYBG 6281, culture G.J.S. 89-60); Mt. Wokomung, Wokomung Base Camp, ca. 8 hr wal NE of Kopinang Village in wet forest dominated by Euphorbiaceae, 05°05’N 59°50’W, 1070 m, Jun-Jul 1989, G.J. Samuels, B.M. Boom, G. Bacchus (NYBG 6318, culture G.J.S. 89-65 = CBS 123970). |
|  |
| PUERTO RICO. 350-400 m, on *Ocotea* sp. twigs, 20 Feb 1996, G.J. Samuels, H.J. Schroers, D.J. Lodge (BPI 744683, culture G.J.S. 96-22 = IMI 370946). |
|  |
| PUERTO RICO. Caribbean National Forest, Luquillo Mountains, Rio Grande, trail to El Toro from rt 186, 650–750 m, on bark of unidentified recently dead tree, 24 Feb 1996, G.J. Samuels, H.-J Schroers, D.J. Lodge (BPI 745542, culture G.J.S. 96-23 = IMI 370947). |
|  |
| VENEZUELA. Sucre State, NW of Irapa, trail between Los Pocitos and the peak of Cerro Humo, on stem of unidentified palm, 12 Jul 1972, K.P. Dumont, R.F. Cain, G.J. Samuels, G. Morillo, J. Farian (NYBG Dumont-VE 4769, culture C.T.R. 72-90). |
|  |
| VENEZUELA. Aragua State, Henri Pittier National Park, Rancho Grande Biological Station, trail to Guacamayo, 1250-1400m, 10°21’N, 67°41’W, on bark of unidentified tree, 04 Dec 1990, G.J. Samuels, B. Hein, S.M. Huhndorf (BPI 842123, culture G.J.S. 90-212 = CBS 134028). |
|  |
| VENEZUELA. Aragua State, Henry Pittier National Park, ca. 20 km above Maracay, on Maracay-Choroni road, on wood of unidentified dead tree, 13 Jul 1971, K.P. Dumont, J.H. Haines, G.J. Samuels (NYBG Dumont-VE 2173, culture C.T.R. 71-281 = CBS 112458). |
|  |
| VENEZUELA. Merida State: Sierra Nevada National Park, above Tabay, Qda. Coromoto, La Mucuy, 08°36’N 71°02’W, ca. 2000 m, on palm fruit, G.J. Samuels et. al. (BPI ??, culture G.J.S. 90-155 = CBS 123966). |
|  |
| VENEZUELA. Bolivar State, The Gran Sabana National Park, 1139 m, on wood of unidentified dead tree, 29 Jun 2009, C. Salgado, Y. Hirooka, YH09-124 (BPI, culture G.J.S. 09-1327 = CBS 134022). |
|  |
| VENEZUELA. 13 km NE of Colonia Tovar on road between Colonia Tovar and El Tigre, Dto. Fed., on bark of unidentified tree, 19 Jul 1972, K.P Dumont, R.F. Cain, G.J. Samuels, B. Manara (NYBG Dumont-VE 6503, culture C.T.R. 72-188 = CBS 134040). |
|  |
| JAPAN. Okutama-gun, on twigs of undetermined plant, 20 Nov 2003, Y. Hirooka TPP-h-229-2 (BPI 882092, culture MAFF 241524). |
|  |
| JAPAN. Miyagi Prefecture, Kenminnomori, Rifu-cho, Miyagi-gun, on twigs of unknown plant, 5 Aug 2004, Y. Hirooka TPP-305-2 (BPI 882109, culture MAFF 241543). |
|  |
| JAPAN. Kanagawa Prefecture, Yamakitagawayosa, Ashigarakami-gun, on bark of undetermined dead tree, 30 Oct 2004, Y. Hirooka TPP-h374-2 (BPI 881926, culture MAFF 241554); on bark of dead *Fagus crenata*, 17 Apr 2005, Y. Hirooka TPP-h-433-2 (BPI 881944, culture MAFF 241563). |
|  |
| JAPAN. Kochi Prefecture, Tosa-cho, on bark of dead tree, 04 Aug 2004, Y. Hirooka TPP-h171-1 (BPI 882162, culture MAFF 241515). |
|  |
| JAPAN. Kochi Prefecture, Tosakitakaido, Tosa-cho, on twigs of Cryptomeria japonica, 04 Aug 2003, Y. Hirooka TPP-h178-2 (BPI 882164, culture MAFF 241517). |
|  |
| JAPAN. Miyagu Prefecture, Akiuootaki, Aki-cho, Taihaku-ku, on twigs of undetermined dead tree, 04 Aug 2004, Y. Hirooka TPP-h292-2 (BPI 882106, culture MAFF 241539). |
|  |
| JAPAN. Tokyo, Sakaigatake, Hahajima, Ogasawara-mura, on bark of unidentified dead tree, 22 Jun 2005, Y. Hirooka TPP-h488-2 (BPI 881951, culture MAFF 241564). |
|  |
| TAIWAN. Taipei County, Jingtung, Jungtung historical trail, on bark, 21 Dec 2003, J.-R. Guu 92122107 (BPI, culture 92122107 = CBS 134038). |
|  |
| TAIWAN. Kaohsiung County, Liou-guei, Shan-ping, on bark of unidentified tree, 10 Mar 2005, J. –R. Guu (BPI, culture 94031007 = CBS 134039) |
|  |
| INDONESIA. North Sulawesi, Eanstern Dumoga-Bone National Park, at confluence of Toraut and Tumpha Rivers, Project Wallace Base Camp, 0°34’N 123°57’E, 211 m, on twig of unidentified tree, Sep-Nov 1985, G.J. Samuels (NYBG 2222A, culture G.J.S. 85-179 = IMI 329113). |
|  |
| AUSTRALIA. Queensland: Atherton City, Davis Creek, on *Acacia celsa*, 2 Feb 2009, A.Y. Rossman, P. Chaverri PC 883 (BPI 879019, culture G.J.S. 09-509). |
|  |
| SWITZERLAND. Horgen Distric: Wadenswill Locality, on *Rubus idaeus* roots, 1911, A. Osterwalder (ex-type culture CBS 113.12 = IMI 113918). |
|  |
| ARGENTINA. Tucuman Province: road to Catamarca, camino Las Lenguas, near to Rio Cochuna, 400 m, on bark of a rotting fallen tree, C. Salgado, A.Y. Rossman, A. Romero (BPI, culture A.R. 4766 = CBS 134035). |
|  |
| UNITED STATES. North Carolina: Macon County, Ellicot Rock Trail, off of Bull Pen road, 35°02’N 83°08’W, 915 m, on bark of living Quercus sp. tree, G.J. Samuels, A.Y. Rossman, Y. Doi (BPI 1107126, culture G.J.S. 90-46 = CBS 134029). |
|  |
| UNITED STATES. Connecticut: New Haven, West Rock Ridge State Park, on bark of dead *Fagus grandifolia*, Oct 2007, R. Marra (BPI 878945, culture A.R. 4499 = CBS 125172). |
