# Supplementary material for: Not as Ubiquitous as We Thought: Taxonomic Crypsis, Hidden Diversity and Cryptic Speciation in the Cosmopolitan Fungus Thelonectria discophora (Nectriaceae, Hypocreales, Ascomycota)
Source: PLoS One. 2013 Oct 18;8(10):e76737. doi: 10.1371/journal.pone.0076737 (PMC3799981; doi:10.1371/journal.pone.0076737)
Supplement: Table S3 — List of molecular markers and descriptive statistics for the six loci used in this study. (DOCX) [file pone.0076737.s010.docx]

Table S3. List of molecular markers and descriptive statistics for the six loci used in this study.

| Locus | Substitution model | Aligned length | Variable  sites (%) | Parsimony informative sites (%) | %GC | Primers | Reference |
| --- | --- | --- | --- | --- | --- | --- | --- |
| *act* | TrN + G | 545 | 66 (12.11) | 43 (7.88) | 56.6 | F 5’TGGCACCACACCTTCTACAATGA3’  R 5’TCCTCCGCTTATTGATATGC3’ | 1 |
| ITS | HKY + G | 661 | 96 (14.52) | 80 (12.10) | 55.3 | F 5’GGAAGTAAAAGTCGTAACAAGG3’  R 5’TCCTCCGCTTATTGATATGC3’ | 2 |
| LSU | TrNef + I | 807 | 44 (5.45) | 35 (4.33) | 53.7 | F 5’ACCCGCTGAACTTAAGC3’  R 5’TCCTGAGGGAAACTTCG3’ | Vilgalys n.d. |
| *tef* | HKY + I + G | 921 | 183 (19.86) | 135 (14.65) | 55.8 | F 5’CATCGAGAAGTTCGAGAAGG3’  R 5’ACHGTRCCRATACCACCRAT3’ | 3 |
| *tub* | TrN + G | 556 | 159 (28.6) | 126 (22.66) | 56.6 | F 5’AACATGCGTGAGATTGTAAGT3’  R 5’TAGTGACCCTTGGCCCAGTTG3’ | 4 |
| *rpb1* | TrNef + I | 646 | 227 (35.13) | 197 (30.5) | 53.4 | F 5’CAYCCWGGYTTYATCAAGAA3’  R 5’CCNGCDATNTCRTTRTCCATRTA3’ | 5 |

1. Samuels GJ, Dodd S, Lu B-S, Petrini O, Schroers H-J, et al. (2006) The *Trichoderma koningii* aggregate species. Stud Mycol 56: 67-133.
2. White TJ, Bruns T, Lee S, Taylor JW (1990) Amplification and direct sequencing of fungal ribosomal RNA genes for phylogenetics. In: PCR Protocols: A guide to methods and Applications. Innis MA, Gelfand DH, Sninsky JJ, White TJ, Eds. Academic Press Inc., New York: 315-322.
3. Carbone I, Kohn LM (1999) A method for designing primer sets for speciation studies in filamentous ascomycetes. Mycologia 85: 612-637.
4. O’Donnell K, Cigelnik E (1997) Two divergent intragenomic rDNA ITS2 types within a monophyletic lineage of the fungus Fusarium are nonorthologous. Mol Phylogenet Evol 7: 103-117.
5. Castlebury LA, Rossman AY, Sung G-H, Hyten AS, Spatafora JW (2004) Multigene phylogeny reveals new lineage for *Stachybotrys chartarum*, the indoor air fungus. Mycol Res 108: 1-9.
